# Transient Accumulation of NO_2_^-^ and N_2_O during Denitrification Explained by Assuming Cell Diversification by Stochastic Transcription of Denitrification Genes

**DOI:** 10.1371/journal.pcbi.1004621

**Published:** 2016-01-05

**Authors:** Junaid Hassan, Zhi Qu, Linda L. Bergaust, Lars R. Bakken

**Affiliations:** 1 Department of Environmental Sciences, Norwegian University of Life Sciences, Ås, Norway; 2 Chemistry, Biotechnology and Food Science, Norwegian University of Life Sciences, Ås, Norway; Ottawa University, CANADA

## Abstract

Denitrifying bacteria accumulate ${\mathrm{NO}}_{2}^{-}$, NO, and N_2_O, the amounts depending on transcriptional regulation of core denitrification genes in response to O_2_-limiting conditions. The genes include *nar*, *nir*, *nor* and *nosZ*, encoding ${\mathrm{NO}}_{3}^{-}$-, ${\mathrm{NO}}_{2}^{-}$-, NO- and N_2_O reductase, respectively. We previously constructed a dynamic model to simulate growth and respiration in batch cultures of *Paracoccus denitrificans*. The observed denitrification kinetics were adequately simulated by assuming a stochastic initiation of *nir*-transcription in each cell with an extremely low probability (0.5% h^-1^), leading to product- and substrate-induced transcription of *nir* and *nor*, respectively, via NO. Thus, the model predicted cell diversification: after O_2_ depletion, only a small fraction was able to grow by reducing ${\mathrm{NO}}_{2}^{-}$. Here we have extended the model to simulate batch cultivation with ${\mathrm{NO}}_{3}^{-}$, i.e., ${\mathrm{NO}}_{2}^{-}$, NO, N_2_O, and N_2_ kinetics, measured in a novel experiment including frequent measurements of ${\mathrm{NO}}_{2}^{-}$. *Pa*. *denitrificans* reduced practically all ${\mathrm{NO}}_{3}^{-}$ to ${\mathrm{NO}}_{2}^{-}$ before initiating gas production. The ${\mathrm{NO}}_{2}^{-}$ production is adequately simulated by assuming stochastic *nar*-transcription, as that for *nirS*, but with a higher probability (0.035 h^-1^) and initiating at a higher O_2_ concentration. Our model assumes that all cells express *nosZ*, thus predicting that a majority of cells have only N_2_O-reductase (A), while a minority (B) has ${\mathrm{NO}}_{2}^{-}$-, NO- and N_2_O-reductase. Population B has a higher cell-specific respiration rate than A because the latter can only use N_2_O produced by B. Thus, the ratio $\frac{\text{B}}{\text{A}}$ is low immediately after O_2_ depletion, but increases throughout the anoxic phase because B grows faster than A. As a result, the model predicts initially low but gradually increasing N_2_O concentration throughout the anoxic phase, as observed. The modelled cell diversification neatly explains the observed denitrification kinetics and transient intermediate accumulations. The result has major implications for understanding the relationship between genotype and phenotype in denitrification research.

## Author Summary

Denitrifiers generally respire O_2_, but if O_2_ becomes limiting, they may switch to anaerobic respiration (denitrification) by producing ${\mathrm{NO}}_{3}^{-}$-, ${\mathrm{NO}}_{2}^{-}$-, NO- and/or N_2_O reductase, encoded by *nar*, *nir*, *nor*, and *nosZ* genes, respectively. Denitrification causes transient accumulation of ${\mathrm{NO}}_{2}^{-}$ and NO/N_2_O emissions, depending on the activity of the four reductases. Denitrifiers lacking *nosZ* produce ~100% N_2_O, whereas organisms with only *nosZ* are net consumers of N_2_O. Full-fledged denitrifiers are equipped with all four reductases, genetic regulation of which determines ${\mathrm{NO}}_{2}^{-}$ accumulation and NO/N_2_O emissions. *Paracoccus denitrificans* is a full-fledged denitrifying bacterium, and here we present a modelling approach to understand its gene regulation. We found that the observed transient accumulation of ${\mathrm{NO}}_{2}^{-}$ and N_2_O can be neatly explained by assuming cell diversification: all cells expressing *nosZ*, while a minority expressing *nar* and *nir*+*nor*. Thus, the model predicts that in a batch culture of this organism, only a minor sub-population is full-fledged denitrifier. The cell diversification is a plausible outcome of stochastic initiation of *nar-* and *nir* transcription, which then becomes autocatalytic by ${\mathrm{NO}}_{2}^{-}$and NO, respectively. The findings are important for understanding the regulation of denitrification in bacteria: product-induced transcription of denitrification genes is common, and we surmise that diversification in response to anoxia is widespread.

## Introduction

The dissimilative reduction of nitrate (${\mathrm{NO}}_{3}^{-}$) to nitrite (${\mathrm{NO}}_{2}^{-}$), nitric oxide (NO), nitrous oxide (N_2_O), and finally to N_2_ (denitrification) is an indispensable process in the nitrogen cycle, returning N to the atmosphere as N_2_. However, denitrification significantly leaks the gaseous intermediates NO and N_2_O, both with serious consequences for the environment. N_2_O catalyses depletion of the stratospheric ozone [1] and causes global warming, contributing ~10% to the anthropogenic climate forcing [2]. Data suggests that since the 1950s, the atmospheric N_2_O has been increasing, and before being photolysed in the stratosphere, the gas persists for an average ~120 years in the troposphere [3]. ~70% of global N_2_O emissions are tentatively attributed to microbial nitrification and denitrification in soils [4], where denitrification, generally, is considered a more dominant source [5].

### To mitigate N_2_O emissions, we need to understand the physiology of denitrifiers

To devise robust strategies for mitigating global N_2_O emissions, a good understanding of its primary source is imperative, i.e., genetics, physiology, and regulatory biology of denitrifiers. Any knowledge of the environmental controllers of N_2_O is incomplete without understanding the causal relationships of such controllers at the physiological level [6].

The biogeochemical models developed for understanding the ecosystem controls of denitrification and N_2_O emissions treat the denitrifying community of soils and sediments as a single homogenous unit with certain characteristic responses to O_2_ and ${\mathrm{NO}}_{3}^{-}$ concentrations [6,7]. Natural denitrifying communities, however, are mixtures of organisms with widely different denitrification regulatory phenotypes [8]. The regulatory response of such mixtures is not necessarily equal to the ‘sum of its components’ because there will be interactions, not the least, via the intermediates NO and ${\mathrm{NO}}_{2}^{-}$. Hence, it is probably a mission impossible to predict the regulatory responses of complex communities based on their phenotypic composition. Nevertheless, investigations of the regulation in model organisms like *Pa*. *denitrificans* provide us with essential concepts, enhancing our ability to understand the regulatory responses of mixed communities and to generate meaningful hypotheses. Thus, future biogeochemical models of N_2_O and NO emissions are expected to have more explicit simulations of the regulatory networks involved, and a first attempt has recently been published [9].

### Simulating the cell diversification in response to impending anoxia to analyse its implications for ${\mathrm{NO}}_{2}^{-}$, N_2_, and N_2_O kinetics

Dynamic modelling has been used to a limited extent to analyse various denitrification phenotypes; for example, to analyse ${\mathrm{NO}}_{3}^{-}$ and ${\mathrm{NO}}_{2}^{-}$ reduction and gas-kinetic data for individual strains [10] and mixtures of selected phenotypes [11]; to model the consequence of competition for electrons between denitrification reductases [12,13]; to investigate the control of O_2_ on denitrification enzymes and inhibition of cytochrome *c* oxidase by NO in *Agrobacterium tumefaciens* [14]; and to examine the effect of copper availability on N_2_O reduction in *Paracoccus denitrificans* [15]. In our previous model [16], we simulated O_2_ and N_2_ kinetics from batch incubations of *Pa*. *denitrificans* [8,17] to test if a postulated cell diversification, driven by stochastic initiation of *nirS*, could explain the N_2_ production kinetics in ${\mathrm{NO}}_{2}^{-}$-supplemented media. The available data also contained ${\mathrm{NO}}_{3}^{-}$-supplemented treatments but ${\mathrm{NO}}_{3}^{-}$ and ${\mathrm{NO}}_{2}^{-}$ were not monitored, and the experiment provided no information about the N_2_O kinetics, except that the concentrations were extremely low (below the detection limit of the thermal conductivity detector used). Recently, a neat dataset was generated from batch incubations supplemented with ${\mathrm{NO}}_{3}^{-}$, with frequent measurements of ${\mathrm{NO}}_{2}^{-}$ and a more sensitive detection of N_2_O by an electron capture detector [18]. That encouraged us to extend our previous model and simulate the cell diversification during transition from oxic to anoxic conditions, targeting the regulation of Nar and *c*Nor/NosZ (N_2_O emissions) in *Pa*. *denitrificans*.

### Regulatory network of denitrification in *Paracoccus denitrificans*

*Pa*. *denitrificans* is a facultative anaerobe capable of reducing ${\mathrm{NO}}_{3}^{-}$ all the way to N_2_:

$${\mathrm{NO}}_{3}^{-}\overset{\text{Nar}}{\to }{\mathrm{NO}}_{2}^{-}\overset{\text{NirS}}{\to }\text{NO}\overset{c\text{Nor}}{\to }{\text{N}}_{2}\text{O}\overset{\text{NosZ}}{\to }{\text{N}}_{2}$$

In response to impending anoxic conditions, the organism sustains respiratory metabolism by producing the membrane-bound cytoplasmic nitrate reductase (Nar), cytochrome *cd*_*1*_ nitrite reductase (NirS), cytochrome *c* dependent nitric oxide reductase (*c*Nor), and nitrous oxide reductase (NosZ). Transcription of the genes encoding these reductases (*narG*, *nirS*, *norBC*, and *nosZ*, respectively) are regulated by the FNR-type proteins FnrP, NarR, and NNR. FnrP contains a 4Fe-4S cluster for sensing O_2_, and NNR harbours a NO-sensing haem; NarR, however, is poorly characterised and is most likely a ${\mathrm{NO}}_{2}^{-}$-sensor [19–21]. All these sensors remain inactive during aerobic growth conditions [19].

#### Transcription of denitrification genes in *Pa*. *denitrificans*

**Fig 1:**
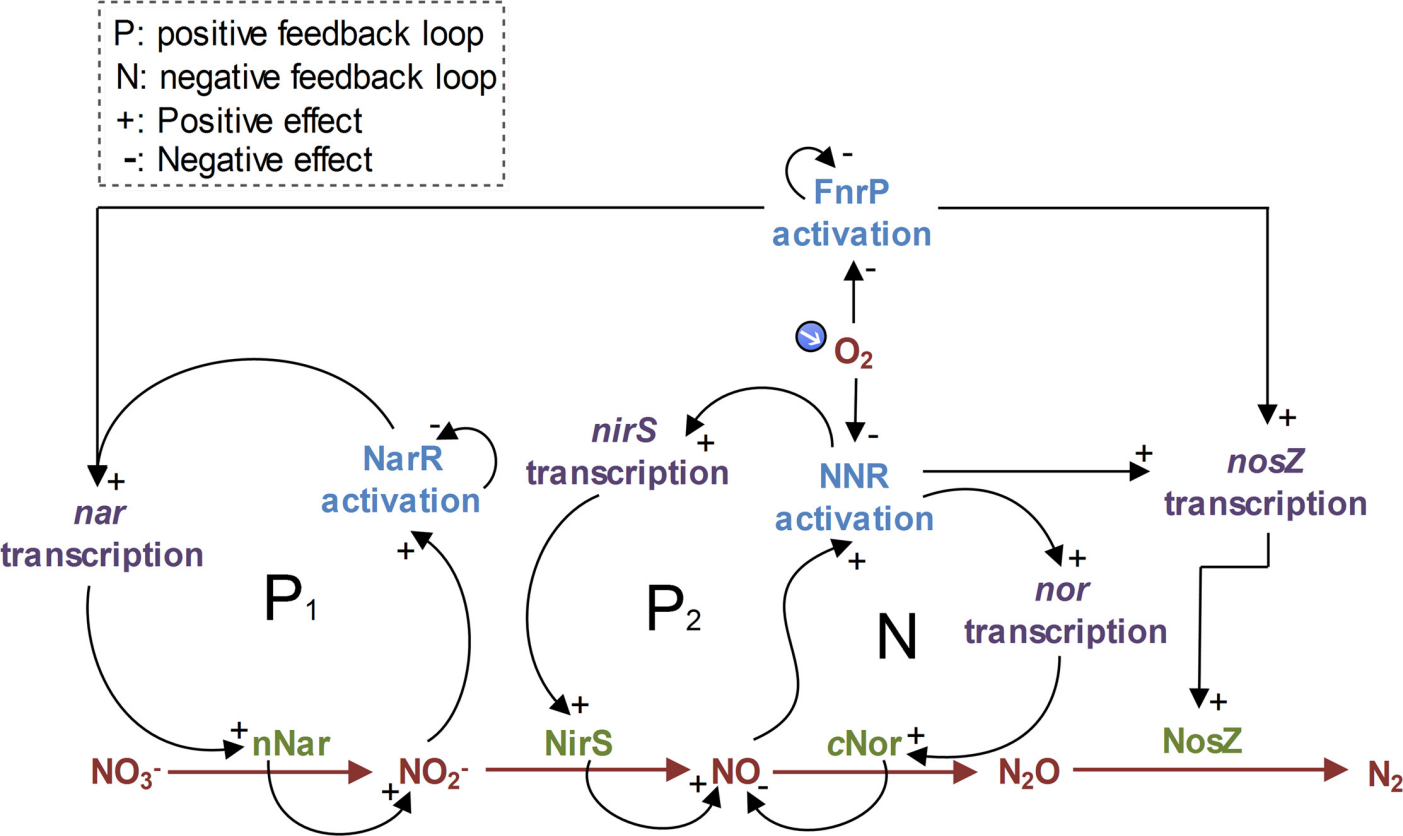
Regulatory network of denitrification in *Pa*. *denitrificans*. The network is driven by four core enzyme-complexes: Nar (transmembrane nitrate reductase encoded by the *narG* gene), NirS (cytochrome *cd*_*1*_ nitrite reductase encoded by *nirS*), *c*Nor (NO reductase encoded by *norBC*), and NosZ (N_2_O reductase encoded by *nosZ*). When anoxia is imminent, the low [O_2_] is sensed by FnrP, which in some interplay with NarR induces *nar* transcription. NarR is activated by ${\mathrm{NO}}_{2}^{-}$; thus once a cell starts producing traces of ${\mathrm{NO}}_{2}^{-}$, *nar* expression becomes autocatalytic (see P_1_). Transcription of *nirS* is induced by NNR, which is activated under anoxic/micro-oxic conditions by NO; thus once traces of NO are produced, the expression of *nirS* also becomes autocatalytic (see P_2_) [20]. The activated P_2_ will also induce *nor* and *nosZ* transcription via NNR. The transcription of *nosZ*, however, can also be induced equally and independently by FnrP [24]. Micromolar concentrations of NO may inactivate both FnrP [25] and NosZ [26]. These observations, however, are ignored for our modelling because *Pa*. *denitrificans* restricts NO to nanomolar levels.

FnrP and NarR facilitate a product-induced transcription of the *nar* genes, and NNR facilitates a product-induced transcription of the *nirS* genes (Fig 1, see P_1_ and P_2_): Low oxygen concentration ([O_2_]) activates the self-regulating FnrP, which induces *nar* transcription in coaction with NarR. The self-regulating NarR was previously assumed to be activated by either ${\mathrm{NO}}_{3}^{-}$ or ${\mathrm{NO}}_{2}^{-}$ [21], but a recent proteomics study indicates that ${\mathrm{NO}}_{2}^{-}$ is the activator [19]. Thus once a cell starts producing traces of ${\mathrm{NO}}_{2}^{-}$, *nar* expression becomes autocatalytic. Transcription of *nirS* is induced by NNR, which is apparently inactivated by O_2_ [22,23], but under anoxic/micro-oxic conditions, NNR is activated by NO. Thus, once traces of NO are produced, the expression of *nirS* also becomes autocatalytic [19,20]. In contrast, *nor* transcription is substrate (NO) induced via NNR while *nosZ* is equally induced by NNR or FnrP [24]. High concentrations of NO may constrain *nar* transcription by inactivating FnrP [25] and, like O_2_, render NosZ dysfunctional by inactivating the Cu_Z_ subunit of the reductase [26], but these observations are ignored in our model because *Pa*. *denitrificans* restricts [NO] to very low levels.

### Entrapment of cells in anoxia: The underlying hypothesis and modelling

Denitrification proteome, once produced in response to an anoxic spell, is likely to linger within the cells under subsequent oxic conditions, ready to be used if anoxia recurs. But the proteome will be diluted by aerobic growth because the transcription of denitrification genes is inactivated under oxic conditions [20]. Hence, a population growing through many generations under fully oxic conditions is expected to undertake *de novo* synthesis of denitrification enzymes when confronted with anoxia. Batch cultivations of such aerobically raised *Pa*. *denitrificans* provided indirect evidence for a novel claim that, in response to anoxia, only a small fraction of the incubated population is able to produce denitrification proteome [8,17,27,28]. Our dynamic modelling of Bergaust *et al*.*’s* [17] ${\mathrm{NO}}_{2}^{-}$-supplemented incubations corroborated this, suggesting that a probabilistic function (specific probability = 0.005 h^-1^) resulting in the recruitment of 3.8–16.1% of all cells to denitrification is adequate to explain the measured N_2_ kinetics [16].

Our model was based on the hypothesis that the entrapment of a large fraction in anoxia is due to a low probability of initiating *nirS* transcription, which in response to O_2_ depletion is possibly mediated through a minute pool of intact NNR, crosstalk with other factors (such as FnrP), unspecific reduction of ${\mathrm{NO}}_{2}^{-}$ to NO by Nar, and/or through non-biologically formed traces of NO found in a ${\mathrm{NO}}_{2}^{-}$-supplemented medium. Regardless of the exact mechanism(s), once *nirS* transcription is initiated, the positive feedback via NO/NNR (Fig 1, see P_2_) would allow the product of a single transcript of *nirS* to induce a subsequent burst of *nirS* transcription. The activated positive feedback will also help induce *nor* and *nosZ* transcription via NNR, rapidly transforming a cell into a full-fledged denitrifier. We further hypothesised that recruitment to denitrification will only be possible as long as a minimum of O_2_ is available because, since *Pa*. *denitrificans* is non-fermentative, the synthesis of first molecules of NirS will depend on energy from aerobic respiration.

The above hypothesis was modelled by segregating the culture into two pools (subpopulations): one for the cells without (N_D−_) and the other with denitrification enzymes (N_D+_). Initially, all cells were N_D−_, growing by consuming O_2_. As [O_2_] fell below a certain threshold, N_D−_ recruited to N_D+_ with a constant probability (h^-1^), assumed to be that of the *nirS* transcriptional activation, and the recruitment halted as O_2_ was completely exhausted, assuming lack of energy (ATP) for enzyme synthesis.

### Underlying assumptions and aims of the present modelling

**Fig 2:**
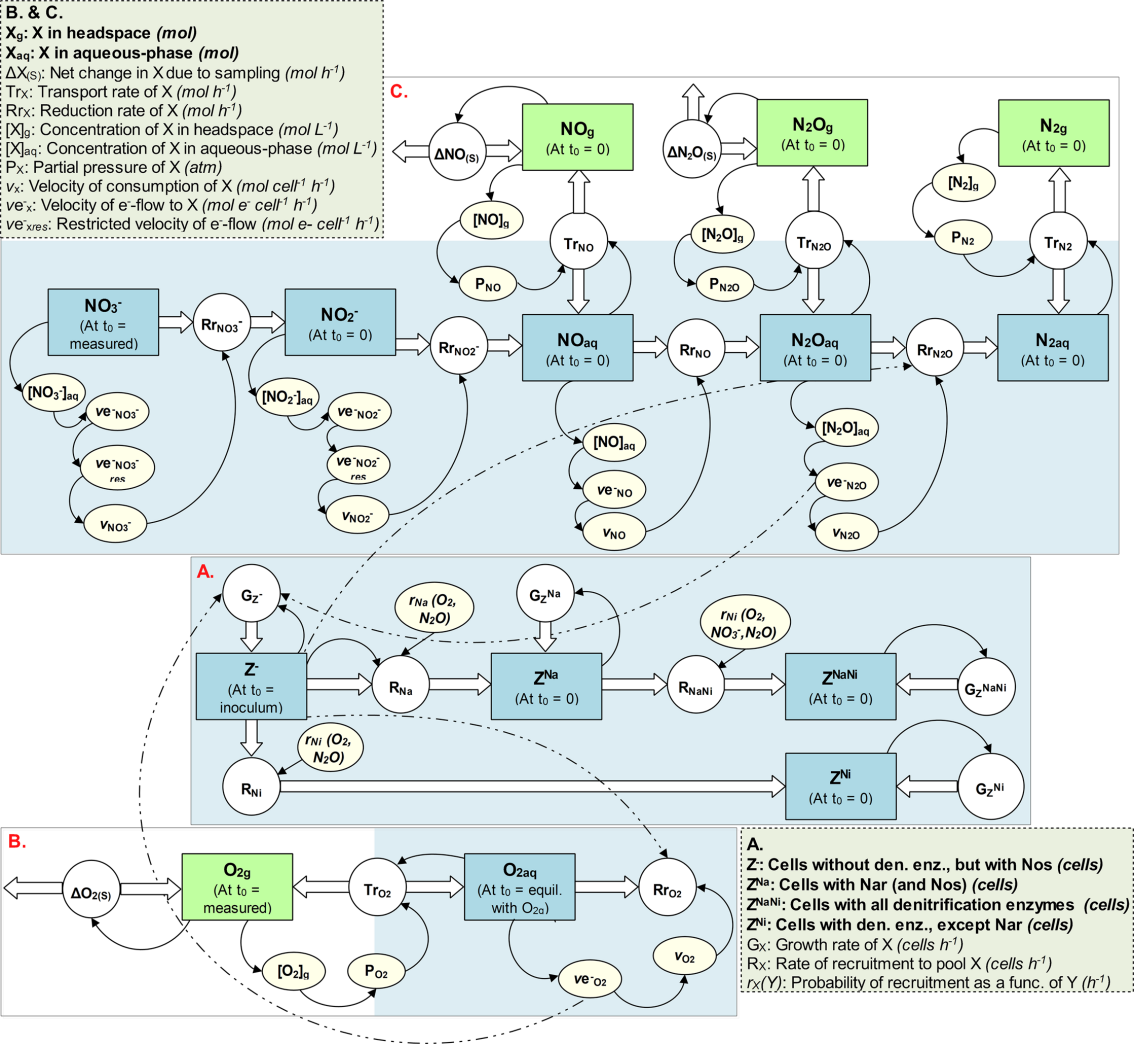
A stock and flow diagram illustrating the model’s structure. **A.** Cell diversification and growth; **B.** O_2_ kinetics; **C.** Denitrification kinetics. The squares represent state variables, the circles the rate of change of the state variables, the edges (thicker arrows) depict flows into or out of the state variables, the shaded ovals auxiliary variables, and the arrows portray mutual dependencies between the variables. All feedback relationships among the three model sectors could not be shown; however, for illustration the feedback relationships of one sub-population (Z^−^) are shown (dashed arrows). Within each square (state variable), t_0_ refers to the initial value.

The present model is an extension of that developed in Hassan *et al*. [16]. Here we have divided the respiring culture into four pools (Fig 2A):

1. Z^−^: cells without Nar, NirS, and *c*Nor
2. Z^Na^: cells with Nar
3. Z^NaNi^: cells with Nar, NirS, and *c*Nor
4. Z^Ni^: cells with NirS and *c*Nor

All these subpopulations are assumed to scavenge O_2_ (if present) and produce NosZ in response to impending anoxia. The latter because the *nosZ* genes are readily induced by the O_2_-sensor FnrP [24].

The Z^−^ pool (Fig 2A) contains the inoculum that grows by aerobic respiration. As [O_2_] falls below a critical threshold [empirically determined, 18], the cells within Z^−^ are assumed to start synthesising Nar with a certain probability and populate the Z^Na^ pool. The aim here is to investigate whether, like for *nirS*, the initiation of *nar* transcription (by some combined activity of FnrP and NarR) can also be explained as a probabilistic phenomenon, quickly differentiating a cell into a full-fledge ${\mathrm{NO}}_{3}^{-}$ scavenger through product (${\mathrm{NO}}_{2}^{-}$) induced transcription via NarR (Fig 1, see P_1_). If so, we were interested to estimate what fraction of the cells is required to adequately simulate the measured data (${\mathrm{NO}}_{2}^{-}$ production), aiming at scrutinising the general assumption that all cells in batch cultures produce Nar in response to impending anoxia.

Next, when [O_2_] is further depleted to another critical threshold [18], the Z^−^ and Z^Na^ cells are assumed to initiate *nirS* transcription with a low per hour probability and, thereby, populate the Z^Ni^ and Z^NaNi^ pools, respectively. As explained above for our previous model, NirS + *c*Nor production is assumed to be *a)* coordinated because the transcription of both *nirS* and *nor* is induced by NO via the NO-sensor NNR (Fig 1), and *b)* stochastic because the initial transcription of *nirS* (paving the way for the autocatalytic expression of NirS and substrate-induced *nor* transcription) happens in the absence of NO or at too low [NO] to be sensed by NNR.

Synthesis of denitrification enzymes requires energy, which all the subpopulations can obtain by respiration only. Hence, the initiation of the autocatalytic expression of *nar* and *nirS* (i.e., recruitment to Z^Na^ and Z^NaNi^/Z^Ni^, respectively, Fig 2A) depends on the availability of the relevant terminal e^-^-acceptor(s) above a critical concentration to sustain a minimum of respiration. For Z^−^, the only relevant e^-^-acceptors are O_2_ and the traces of N_2_O produced by Z^Ni^ and Z^NaNi^. The same applies For Z^Na^, but in addition, this subpopulation can also obtain energy by reducing ${\mathrm{NO}}_{3}^{-}$, if present. In our previous model [16], we assumed that recruitment to denitrification was sustained by energy from O_2_-respiration only; not ${\mathrm{NO}}_{3}^{-}$ because we simulated ${\mathrm{NO}}_{2}^{-}$-supplemented treatments, and not by N_2_O because we naively assumed that the pool of this e^-^-acceptor was insignificant (N_2_O concentrations were below the detection limit of the system used for those experiments). However, the present model assumes that the recruitment from Z^−^ to Z^Na^ and Z^−^ to Z^Ni^ is sustained by both O_2_- and N_2_O-reduction, and the recruitment from Z^Na^ to Z^NaNi^ is sustained by O_2_-, N_2_O- and ${\mathrm{NO}}_{3}^{-}$-reduction, when above a critical minimum ($v{\text{e}}_{min}^{-}$). The default value for $v{\text{e}}_{min}^{-}$ was set to an arbitrary low value (= 0.44% of the maximum e^-^-flow rate to O_2_), and we have investigated the consequences of increasing, decreasing, and setting $v{\text{e}}_{min}^{-}$ = 0.

The expressions of *nar* and *nirS* + *nor* (recruitments to Z^Na^ and Z^NaNi^/Z^Ni^, respectively, Fig 2A) are modelled as instantaneous discrete-events in each cell, thus ignoring the time-lag from the initiation of gene transcription till the cell is fully equipped with the reductase(s) in question. That is because the lag observed between the emergence of denitrification gene transcripts and the subsequent gas products suggests that the synthesis of denitrification enzymes takes less than half an hour [17,18], which is negligible for our purposes here.

The main purpose of the present modelling is to investigate if a full-fledged model, including all four functional denitrification reductases, could adequately simulate the observed kinetics and stoichiometry of denitrification products [18]. These cultures reduced all available ${\mathrm{NO}}_{3}^{-}$ to ${\mathrm{NO}}_{2}^{-}$ prior to the onset of gas production and accumulated traces of N_2_O throughout the anoxic phase, as illustrated in S1 Fig In particular, we were interested to investigate the ${\mathrm{NO}}_{2}^{-}$ kinetics, controlled by *nar*- and *nirS* transcription, and to test if the peculiar N_2_O kinetics (low, but increasing concentrations throughout the anoxic phase) could be explained by our modelled cell diversification.

## Materials and Methods

### An overview of the modelled experiment

#### Batch incubation

Qu [18] incubated *Pa*. *denitrificans* (DSM-413) at 20°C using 50 mL Sistrom’s [29] medium in 120 mL gas-tight vials. Either succinate or butyrate (5 mM) was used as the main carbon source, enough to secure consumption of all available e^-^-acceptors. After distribution of the medium, each vial was loaded with a magnetic stirring bar, sterilised through autoclaving, supplemented with 2 mM KNO_3_, and was tightly sealed. To remove O_2_ and N_2_ from the headspace, the headspace air was evacuated and replaced by helium (He) through several cycles of evacuation and He-filling (He-washing). Some vials were supplemented with oxygen to reach 7 vol.% O_2_ in headspace (treatment designated 7% O_2_). The remaining vials received no O_2_ (designated 0% O_2_, although there were traces of O_2_ present despite the He washing). For each treatment (i.e., C source and initial O_2_), there were three replicates, and each vial was inoculated with 2.2×10^8^ aerobically grown cells.

#### ${\mathrm{NO}}_{2}^{-}$ and gas measurement

Gases (CO_2_, O_2_, NO, N_2_O, and N_2_) were monitored by frequent sampling of the headspace, using an improved version of the robotised incubation system [30]. In short, the system draws gas samples from the headspace (peristaltic pumping) via the septum pierced by a needle, filling three loops used for injecting samples into the two GC columns and the chemiluminescence NO analyser. The sample drawn is replaced by He (reversing the peristaltic pump), thus securing ~1 atm pressure. The primary improvements of the new system are a more sensitive detection of N_2_O (by an electron capture detector), lower sampling volumes (~1 mL), and lower leaks of O_2_ and N_2_ through the sampling system (4 nmol O_2_ and 12 nmol N_2_ per sampling, which is ~20% of that for the old system).

To extract samples for measuring ${\mathrm{NO}}_{2}^{-}$ without tampering the original vials, identical (parallel) vials were prepared for each treatment. Using sterile syringes, samples of 0.1 mL were regularly drawn from the liquid-phase of the parallel vials and immediately analysed for ${\mathrm{NO}}_{2}^{-}$.

Results for one of the treatments are shown in S1 Fig, illustrating the complete reduction of ${\mathrm{NO}}_{3}^{-}$ to ${\mathrm{NO}}_{2}^{-}$ prior to the onset of significant N-gas production. In previous experiments [17], N_2_O concentrations were below the detection limit of the system, but thanks to the new system, the N_2_O kinetics were monitored with a reasonable precision.

### The model

The model is constructed in Vensim DSS 6.2 Double Precision (Ventana Systems, inc. http://vensim.com/) using techniques from the field of system dynamics [31].

#### Cell diversification and growth

The respiring population is divided into four subpopulations, according to their reductases (Fig 2A): 1) Z^−^: cells without Nar, NirS, and *c*Nor; 2) Z^Na^: cells with Nar; 3) Z^NaNi^: cells with Nar, NirS, and *c*Nor; and 4) Z^Ni^: cells with NirS and *c*Nor. All the subpopulations are assumed to equally respire O_2,_ if present, and express *nosZ* in response to oxygen depletion [24]. Z^−^ contains the inoculum (= 2.2×10^8^ cells) that grows by aerobic respiration. As O_2_ is depleted, the Z^−^ cells populate the other pools by producing Nar and/or NirS + *c*Nor.

The recruitment from Z^−^ to Z^Na^ (R_Na_, Fig 2A) takes place first:

$$R_{\mathrm{Na}}=Z^{-}\times r_{Na}(O_{2},N_{2}O)$$ (1)

(cells h^-1^)

where *r*_*Na*_ *(O*_*2*_,*N*_*2*_ *O)* is a conditional specific probability (h^-1^) for any Z^−^ cell to initiate *nar* transcription (quickly transforming it into a ${\mathrm{NO}}_{3}^{-}$ scavenger through autocatalytic gene expression, see Fig 1, P_1_):

$$\begin{array}{l} r_{Na}(O_{2},N_{2}O)=\\ \mathrm{IF}\;{\left[{\text{O}}_{2}\right]}_{\mathrm{aq}}<{\left[{\text{O}}_{2}\right]}_{na}\;\mathrm{AND}\;\left(v{\text{e}}_{{}_{{\text{O}}_{2}}}^{-}+0.5\times v{\text{e}}_{{}_{{\text{N}}_{2}\text{O}}}^{-}\right)>v{\text{e}}_{min}^{-}\\ \mathrm{THEN}\;{\text{r}}_{\text{Na}}\\ \mathrm{ELSE}\;0 \end{array}$$ (2)

(h^-1^)

where r_Na_ (h^-1^) is a constant specific probability for a cell to initiate *nar* transcription once O_2_ concentration in the aqueous-phase ([O_2_]_aq_, mol L^-1^) falls below a critical concentration ([O_2_]_*na*_), empirically determined as the [O_2_]_aq_ (= 4.75×10^−5^ mol L^-1^) at the outset of ${\mathrm{NO}}_{2}^{-}$ accumulation in the medium [18]. The second condition for a cell to produce first molecules of Nar is a minimum of e^-^-flow to an e^-^-acceptor ($v{\text{e}}_{min}^{-}$, mol e^-^ cell^-1^ h^-1^), assumed to generate minimum ATP required for protein synthesis. $v{\text{e}}_{{}_{{\text{O}}_{2}}}^{-}$and $v{\text{e}}_{{}_{{\text{N}}_{2}\text{O}}}^{-}$ (mol e^-^ cell^-1^ h^-1^) are the cell-specific velocities of e^-^-flow to O_2_ and N_2_O, respectively. The latter is weighed down by 0.5 because mole ATP per mole e^-^ transferred to $NO_{x}^{-}$/NO_x_ is lower for denitrification than for aerobic respiration [17,20]. For a Z^−^ cell, $v{\text{e}}_{{\mathrm{NO}}_{2}^{-}}^{-}$ and $v{\text{e}}_{{}_{\text{NO}}}^{-}$ are not considered here, since such a cell is assumed to have no NirS and *c*Nor.

The fraction of the cells that successfully produces Nar (F_Na_) is calculated based on the integral of the recruitment (Eq 1):

$${\text{F}}_{\text{Na}}=1-e^{-r_{Na}\times t_{Na}}$$ (3)

(dimensionless)

where *t*_*Na*_ is the time-window available for the recruitment. In theory, *t*_*Na*_ is the time-period when ${{[\text{O}}_{2}]}_{\mathrm{aq}}<{{[\text{O}}_{2}]}_{na}\;\mathrm{AND}\;(v{\text{e}}_{{}_{{\text{O}}_{2}}}^{-}+0.5\times v{\text{e}}_{{}_{{\text{N}}_{2}\text{O}}}^{-})>v{\text{e}}_{min}^{-}$ (Eq 2). Since the e^-^-flow to N_2_O started after all ${\mathrm{NO}}_{3}^{-}$ had been reduced to ${\mathrm{NO}}_{2}^{-}$ (S1 Fig), the recruitment based on $v{\text{e}}_{{}_{{\text{N}}_{2}\text{O}}}^{-}$would be inconsequential for the simulated (and measured) ${\mathrm{NO}}_{2}^{-}$ kinetics. Therefore, to calculate the functional F_Na_ actually responsible for producing ${\mathrm{NO}}_{2}^{-}$, we ignored the N_2_O-sustained recruitment, thus considering *t*_*Na*_ to be the time when ${{[\text{O}}_{2}]}_{\mathrm{aq}}<{{[\text{O}}_{2}]}_{na}\;\mathrm{AND}\;v{\text{e}}_{{}_{{\text{O}}_{2}}}^{-}>v{\text{e}}_{min}^{-}$.

Next, the cells within Z^Na^ and Z^−^ are recruited to Z^NaNi^ and Z^Ni^ (R_NaNi_ and R_Ni_, respectively, Fig 2A), as they are assumed to stochastically initiate *nirS* transcription, paving the way for NO/NNR mediated autocatalytic expression of *nirS* + *nor* (Fig 1). In principle, the rates of both these recruitments are modelled as that of the recruitment from Z^−^ to Z^Na^ (Eqs 1 and 2): *a)* Both trigger as O_2_ falls below another critical concentration ([O_2_]_*ni*_), low enough to activate NNR to induce *nirS* transcription; [O_2_]_*ni*_ (= 1.16×10^−5^ mol L^-1^) is empirically determined as the O_2_ concentration at the outset of NO accumulation [18]. *b)* Both continue as long as a minimum of e^-^-flow to the relevant terminal e^-^-acceptor is possible, sustaining the respiratory metabolism to generate ATP for protein synthesis:

$${\text{R}}_{\mathrm{NaNi}}=Z^{\mathrm{Na}}\times r_{Ni}(O_{2},NO_{3}^{-},N_{2}O)$$ (4)

(cells h^-1^)

$$\begin{array}{l} r_{Ni}(O_{2},NO_{3}^{-},N_{2}O)=\\ \mathrm{IF}\;{\left[{\text{O}}_{2}\right]}_{\mathrm{aq}}<{\left[{\text{O}}_{2}\right]}_{ni}\;\mathrm{AND}\;\left(v{\text{e}}_{{}_{{\text{O}}_{2}}}^{-}+0.5\times v{\text{e}}_{{\mathrm{NO}}_{3}^{-}}^{-}+0.5\times v{\text{e}}_{{}_{{\text{N}}_{2}\text{O}}}^{-}\right)>v{\text{e}}_{min}^{-}\\ \mathrm{THEN}\;{\text{r}}_{\mathrm{Ni}}\\ \mathrm{ELSE}\;0 \end{array}$$ (5)

(h^-1^)

where r_Ni_ is a constant specific probability (h^-1^) for the initiation of *nirS* transcription. $v{\text{e}}_{{\mathrm{NO}}_{3}^{-}}^{-}$ and $v{\text{e}}_{{}_{{\text{N}}_{2}\text{O}}}^{-}$ are multiplied with 0.5 for the same reasons as described for Eq 2.

The recruitment from Z^−^ to Z^Ni^ (R_Ni_, Fig 2A) is modelled as a product of Z^−^ and a conditional specific probability, *r*_*Ni*_ *(O*_*2*_,*N*_*2*_ *O)*, which is different from Eq 5 only in that $v{\text{e}}_{{\mathrm{NO}}_{3}^{-}}^{-}$ is omitted, since Z^−^ do not possess Nar:

$${\text{R}}_{\mathrm{Ni}}=Z^{-}\times r_{Ni}(O_{2},N_{2}O)$$ (6)

(cells h^-1^)

$$\begin{array}{l} r_{Ni}(O_{2},N_{2}O)=\\ \mathrm{IF}\;{\left[{\text{O}}_{2}\right]}_{\mathrm{aq}}<{\left[{\text{O}}_{2}\right]}_{ni}\;\mathrm{AND}\;\left(v{\text{e}}_{{}_{{\text{O}}_{2}}}^{-}+0.5\times v{\text{e}}_{{}_{{\text{N}}_{2}\text{O}}}^{-}\right)>v{\text{e}}_{min}^{-}\\ \mathrm{THEN}\;{\text{r}}_{\mathrm{Ni}}\\ \mathrm{ELSE}\;0 \end{array}$$ (7)

(h^-1^)

The fraction that successfully produced NirS + *c*Nor (F_Ni_) is calculated based on the integral of R_NaNi_ and R_Ni_:

$$F_{\mathrm{Ni}}=\left(1-e^{-r_{Ni}\times t_{NaNi}}\right)\times F_{\mathrm{Na}}+\left(1-e^{-r_{Ni}\times t_{Ni}}\right)\times \left(1-F_{\mathrm{Na}}\right)$$ (8)

(dimensionless)

where *t*_*NaNi*_ is the duration of the recruitment from Z^Na^ to Z^NaNi^, i.e., when ${{[\text{O}}_{2}]}_{\text{aq}}<{{[\text{O}}_{2}]}_{ni}\;\mathrm{AND}\;(v{\text{e}}_{{}_{O_{2}}}^{-}+0.5\times v{\text{e}}_{{\mathrm{NO}}_{3}^{-}}^{-}+0.5\times v{\text{e}}_{{}_{{\text{N}}_{2}\text{O}}}^{-})>v{\text{e}}_{min}^{-}$ (Eqs 4 and 5), F_Na_ is the fraction recruited to the pool of Nar positive cells (Z^Na^, Eq 3), and *t*_*Ni*_ is the duration of the recruitment from Z^−^ to Z^Ni^, i.e., when ${{[\text{O}}_{2}]}_{\text{aq}}<{{[\text{O}}_{2}]}_{ni}\;\mathrm{AND}\;(v{\text{e}}_{{}_{O_{2}}}^{-}+0.5\times v{\text{e}}_{{}_{{\text{N}}_{2}\text{O}}}^{-})>v{\text{e}}_{min}^{-}$ (Eqs 6 and 7).

Each of the populations will grow depending on the rates of e^-^-flow to the various e^-^-acceptors they are able to use:

$${\text{G}}_{{\text{Z}}^{-}}={\text{Z}}^{-}\times \left({\text{Ye}}_{{}_{O_{2}}}^{-}\times v{\text{e}}_{{}_{O_{2}}}^{-}+{\text{Ye}}_{{}_{NO_{x}}}^{-}\times v{\text{e}}_{{}_{{\text{N}}_{2}\text{O}}}^{-}\right)$$ (9)

$${\text{G}}_{{\text{Z}}^{\text{Na}}}={\text{Z}}^{\text{Na}}\times \left[{\text{Ye}}_{{}_{O_{2}}}^{-}\times v{\text{e}}_{{}_{O_{2}}}^{-}+{\text{Ye}}_{{}_{NO_{x}}}^{-}\left(v{\text{e}}_{{\mathrm{NO}}_{3}^{-}res}^{-}+v{\text{e}}_{{}_{{\text{N}}_{2}\text{O}}}^{-}\right)\right]$$ (10)

$$G_{Z^{\mathrm{NaNi}}}=Z^{\mathrm{NaNi}}\times \left[{\text{Ye}}_{{}_{O_{2}}}^{-}\times v{\text{e}}_{{}_{O_{2}}}^{-}+{\text{Ye}}_{{}_{NO_{x}}}^{-}\left(v{\text{e}}_{{\text{NO}}_{3}^{-}res}^{-}+v{\text{e}}_{{\mathrm{NO}}_{2}^{-}res}^{-}+v{\text{e}}_{{}_{\text{NO}}}^{-}+v{\text{e}}_{{}_{{\text{N}}_{2}\text{O}}}^{-}\right)\right]$$ (11)

$$G_{Z^{\mathrm{Ni}}}={\text{Z}}^{\text{Ni}}\times \left[{\text{Ye}}_{{}_{O_{2}}}^{-}\times v{\text{e}}_{{}_{O_{2}}}^{-}+{\text{Ye}}_{{}_{NO_{x}}}^{-}\left(v{\text{e}}_{{\mathrm{NO}}_{2}^{-}res}^{-}+v{\text{e}}_{{}_{\text{NO}}}^{-}+v{\text{e}}_{{}_{{\text{N}}_{2}\text{O}}}^{-}\right)\right]$$ (12)

(cells h^-1^)

where ${\text{Ye}}_{{}_{X}}^{-}$ (cells mol^-1^ e^-^ to X = O_2_ or $NO_{x}^{-}$/NO_x_) is the growth yield determined under the actual experimental conditions, and $v{\text{e}}_{{}_{\text{X}}}^{-}$ (mol e^-^ cell^-1^ h^-1^) is the cell-specific velocity of e^-^-flow to X (O_2_ or $NO_{x}^{-}$/NO_x_), which depends on the concentration of the e^-^-acceptor (see Eqs 17, 20 and 28). For ${\mathrm{NO}}_{3}^{-}$ and ${\mathrm{NO}}_{2}^{-}$, a restricted velocity ($v{\text{e}}_{NO_{x}^{-}res}^{-}$) is used so that when electrons flow to O_2_, ${\mathrm{NO}}_{3}^{-}$, and ${\mathrm{NO}}_{2}^{-}$ simultaneously, the total *v*e^−^ per cell does not exceed the maximum electrons that the TCA cycle ($v{\text{e}}_{max\text{TCA}}^{-}$) can deliver per hour (see Eqs 21 and 22).

#### O_2_ kinetics

O_2_ is initially present in the headspace ($O_{2_{g}}$, mol, initialised according to the experiment, see Table 1) but is transported to the liquid-phase ($O_{2_{\mathrm{aq}}}$) due to its consumption therein (Fig 2B). The transport rate ($Tr_{O_{2}}$) is modelled according to Molstad *et al*. [30]:

$${\text{Tr}}_{{\text{O}}_{2}}={\text{k}}_{\text{t}}\;\left({\text{k}}_{\text{H}({\text{O}}_{2})}\times {\text{P}}_{{\text{O}}_{2}}-{\left[{\text{O}}_{2}\right]}_{\text{LP}}\right)$$ (13)

(mol h^-1^)

where k_t_ (L h^-1^) is the empirically determined coefficient for the transport of gas between the headspace and the liquid, ${\text{k}}_{\text{H}({\text{O}}_{2})}$ (mol L^-1^ atm^-1^) is the solubility of O_2_ in water at 20°C, ${\text{P}}_{{\text{O}}_{2}}$ (= [O_2_]_g_ × R × T, atm) is the partial pressure of O_2_ in the headspace, and [O_2_]_aq_ (mol L^-1^) is the O_2_ concentration in the liquid $\left({{[\text{O}}_{2}]}_{\text{aq}}=\frac{{\text{O}}_{2}{}_{{}_{\text{aq}}}}{{\text{Vol}}_{\text{aq}}}\right)$.

**Table 1:** Simulated experiment [18].

Batch	C-source	$O_{2}{}_{{}_{g}}(t_{0})$ (vol.%)*	${\mathrm{NO}}_{3}^{-}(t_{0})$ (mM)	Replicates
1	Butyrate	~0	2	3
2	Butyrate	7	2	3
3	Succinate	~0	2	3
4	Succinate	7	2	3

*Target values for initial O_2_ concentrations in the headspace (vol.%). ~0 means that the intended concentration should be zero, but there were detectable traces of O_2_, despite several cycles of evacuation and He-flushing of the headspace.

In addition, the model simulates the changes in $O_{2_{g}}$ due to sampling. The robotised incubation system used monitors gas concentrations by sampling the headspace, where each sampling alters the concentrations in a predictable manner: a fraction of $O_{2_{g}}$ is removed and replaced by He (dilution), but the sampling also results in a marginal leakage of O_2_ through the tubing and membranes in the injection system. The net change in $O_{2_{g}}$ (ΔO_2(S)_) as a result of each sampling is calculated as:

$${\Delta \text{O}}_{2\left(\text{S}\right)}=\frac{{\text{O}}_{2_{\text{leak}}}-{\text{O}}_{2_{g}}\times \text{D}}{{\text{t}}_{\text{s}}}$$ (14)

(mol h^-1^)

where ${\text{O}}_{2_{\text{leak}}}$ (mol vial^-1^) is the O_2_-leakage into the headspace, D (dilution) is the fraction of each headspace gas removed and replaced by equal amount of He, and t_s_ (h) is the time taken to complete each sampling. ΔO_2(S)_ is negative if $O_{2_{g}}$ is high and marginally positive at very low oxygen concentrations.

O_2_ in the liquid-phase ($O_{2_{\mathrm{aq}}}$, mol, Fig 2B) is initialised by assuming equilibrium with $O_{2_{g}}$ at the time of inoculation (${\text{O}}_{2}{}_{{}_{\mathrm{aq}}}{(\text{t}}_{0})={\text{P}}_{{\text{O}}_{2}}\times {\text{k}}_{\text{H}\left({\text{O}}_{2}\right)}\times {\text{Vol}}_{\mathrm{aq}}$). The dynamics of $O_{2_{\mathrm{aq}}}$ are modelled as a function of transport between the headspace and the liquid (${\text{Tr}}_{{\text{O}}_{2}}$, Eq 13) and its reduction rate (${\text{Rr}}_{{\text{O}}_{2}}$, mol h^-1^):

$$\frac{{\text{d}(\text{O}}_{2_{\text{aq}}})}{\text{dt}}={\text{Tr}}_{{\text{O}}_{2}}-R{\text{r}}_{{\text{O}}_{2}}$$ (15)

$${\text{Rr}}_{{\text{O}}_{2}}=\left(Z^{-}+{\text{Z}}^{\text{Na}}+{\text{Z}}^{\text{NaNi}}+{\text{Z}}^{\text{Ni}}\right)\times v_{{\text{O}}_{2}}$$ (16)

(mol h^-1^)

where Z^−^, Z^Na^, Z^NaNi^, and Z^Ni^ (cells) are all the sub-populations present (described above); thus, we assume that all cells have the same potential to consume O_2_. $v_{O}{}_{{}_{2}}$ (mol cell^-1^ h^-1^) is the cell-specific velocity of O_2_ consumption, obtained by the velocity of e^-^-flow to O_2_ $\left(v{\text{e}}_{{}_{{\text{O}}_{2}}}^{-},\frac{1\;\text{mol}{\text{O}}_{2}}{4\;\text{mol}\;{\text{e}}^{-}}\right)$, where $v{\text{e}}_{{}_{{\text{O}}_{2}}}^{-}$ is modelled as a Michaelis-Menten function of oxygen concentration:

$$v{\text{e}}_{{\text{O}}_{2}}^{-}=\frac{v{\text{e}}_{max{\text{O}}_{2}}^{-}\times {{[\text{O}}_{2}]}_{\mathrm{aq}}}{{\text{K}}_{m{\text{O}}_{2}}+{{[\text{O}}_{2}]}_{\mathrm{aq}}}$$ (17)

(mol e^-^ cell^-1^ h^-1^)

where $v{\text{e}}_{max{\text{O}}_{2}}^{-}$ (mol e^-^ cell^-1^ h^-1^) is the maximum velocity of e^-^-flow to O_2_ per cell (determined under the actual experimental conditions), [O_2_]_aq_ (mol L^-1^) is the O_2_ concentration in the liquid-phase, and ${\text{K}}_{m{\text{O}}_{2}}$ (mol L^-1^) is the half-saturation constant for O_2_ reduction.

#### Denitrification kinetics

The **${\mathrm{NO}}_{3}^{-}$** and ${\mathrm{NO}}_{2}^{-}$ pools (mol, Fig 2C) are initialised according to the experiment (Table 1; ${\mathrm{NO}}_{2}^{-}$ = 0). The kinetics of these nitrogen oxyanions ($NO_{x}^{-}$) are modelled as:

$$\frac{{\text{d(NO}}_{3}^{-})}{\text{dt}}=-R{\text{r}}_{{\text{NO}}_{3}^{-}}=-\left({\text{Z}}^{\text{Na}}+{\text{Z}}^{\text{NaNi}}\right)\times v_{{\text{NO}}_{3}^{-}}$$ (18)

$$\frac{{\text{d(NO}}_{2}^{-})}{\text{dt}}={\text{Rr}}_{{\text{NO}}_{3}^{-}}-R{\text{r}}_{{\text{NO}}_{2}^{-}}=R{\text{r}}_{{\text{NO}}_{3}^{-}}-\left({\text{Z}}^{\text{NaNi}}+{\text{Z}}^{\text{Ni}}\right)\times v_{{\text{NO}}_{2}^{-}}$$ (19)

(mol h^-1^)

where $R{\text{r}}_{{\text{NO}}_{x}^{-}}$ (mol h^-1^) is the reduction rate, Z^Na^ + Z^NaNi^ (cells) is the total number of cells with Nar, Z^NaNi^ + Z^Ni^ (cells) is the total NirS active population, and $v_{NO_{x}^{-}}$ (mol cell^-1^ h^-1^) is the cell-specific velocity of $NO_{x}^{-}$ consumption, obtained by the velocity of e^-^-flow to $NO_{x}^{-}$ $\left(\frac{1\;{\text{mol NO}}_{3}^{-}}{2\;\text{mol}\;{\text{e}}^{-}}\;\text{and}\;\frac{1\;{\text{mol NO}}_{2}^{-}}{1\;\text{mol}\;{\text{e}}^{-}}\right)$. The latter is modelled as a Michaelis-Menten function of $NO_{x}^{-}$concentration:

$$v{\text{e}}_{NO_{x}^{-}}^{-}=\frac{v{\text{e}}_{maxNO_{x}^{-}}^{-}\times {\left[NO_{x}^{-}\right]}_{\text{aq}}}{{\text{K}}_{mNO_{x}^{-}}+{\left[NO_{x}^{-}\right]}_{\text{aq}}}$$ (20)

(mol e^-^ cell^-1^ h^-1^)

where $v{\text{e}}_{maxNO_{x}^{-}}^{-}$ (mol e^-^ cell^-1^ h^-1^) is the maximum velocity of e^-^-flow to $NO_{x}^{-}$ per cell (determined under the actual experimental conditions), ${\left[NO_{x}^{-}\right]}_{\text{aq}}$ (mol L^-1^) is the $NO_{x}^{-}$ concentration in the aqueous-phase, and ${\text{K}}_{mNO_{x}^{-}}$ (mol L^-1^) is the half-saturation constant for $NO_{x}^{-}$ reduction.

The velocity of ${\mathrm{NO}}_{3}^{-}$ and ${\mathrm{NO}}_{2}^{-}$ consumption had to be restricted ($v{\text{e}}_{NO_{x}^{-}res}^{-}$) to ensure that when electrons flow to O_2_, ${\mathrm{NO}}_{3}^{-}$ and ${\mathrm{NO}}_{2}^{-}$ simultaneously, the total *v*e^−^ per cell does not exceed an estimated maximum delivery of electrons from the TCA cycle ($v{\text{e}}_{max\text{TCA}}^{-}$). In competition for electrons, O_2_ is prioritised [20], followed by ${\mathrm{NO}}_{3}^{-}$ and ${\mathrm{NO}}_{2}^{-}$, respectively [18]:

$$v{\text{e}}_{{\text{NO}}_{3}^{-}res}^{-}=\mathrm{Min}\;\left(v{\text{e}}_{{\mathrm{NO}}_{3}^{-}}^{-},\left(v{\text{e}}_{max\text{TCA}}^{-}-v{\text{e}}_{{\text{O}}_{2}}^{-}\right)\right)$$ (21)

$$v{\text{e}}_{{\text{NO}}_{2}^{-}res}^{-}=\mathrm{Min}\;\left(v{\text{e}}_{{\mathrm{NO}}_{2}^{-}}^{-},\left(v{\text{e}}_{max\text{TCA}}^{-}-v{\text{e}}_{{\text{O}}_{2}}^{-}-v{\text{e}}_{{\mathrm{NO}}_{3}^{-}res}^{-}\right)\right)$$ (22)

(mol e^-^ cell^-1^ h^-1^)

where $v{\text{e}}_{{\text{NO}}_{3}^{-}res}^{-}$ is the realised e^-^-flow to ${\mathrm{NO}}_{3}^{-}$, limited either by available ${\mathrm{NO}}_{3}^{-}$ or the availability of electrons (due to competition with O_2_); $v{\text{e}}_{{\text{NO}}_{2}^{-}res}^{-}$is the realised e^-^-flow to ${\mathrm{NO}}_{2}^{-}$. Such competition for electrons was not implemented for $v{\text{e}}_{{}_{\text{NO}}}^{-}$ and $v{\text{e}}_{{}_{{\text{N}}_{2}\text{O}}}^{-}$ because at the onset of NO-, N_2_O- and N_2_ production, the total velocity of e^-^-flow to all available e^-^-acceptors (as predicted by the enzyme kinetics alone) never exceeded $v{\text{e}}_{max\text{TCA}}^{-}$.

Gas consumption and production takes place in the aqueous phase, but the gases are transported between aqua and the headspace depending on their concentrations in the two phases. Each gas in aqua, X_aq_ (molN, Fig 2C), is modelled as a function of production, consumption (not applicable to N_2_), and the net transport, where N_2_O_aq_ and $N_{2}{}_{{}_{\mathrm{aq}}}$ are initialised with zero, and NO_aq_ is initialised with a negligible 1×10^−25^ mol to avoid division by zero (in Eq 28).

$$\frac{d{(\text{NO}}_{\mathrm{aq}})}{\mathrm{dt}}=R{\text{r}}_{{\text{NO}}_{2}^{-}}-R{\text{r}}_{\text{NO}}+T{\text{r}}_{\text{NO}}$$ (23)

$$\frac{d{(\text{N}}_{2}{\text{O}}_{\text{aq}})}{\mathrm{dt}}=R{\text{r}}_{\text{NO}}-R{\text{r}}_{{\text{N}}_{2}\text{O}}+T{\text{r}}_{{\text{N}}_{2}\text{O}}$$ (24)

$$\frac{d{(\text{N}}_{2_{\text{aq}}})}{\mathrm{dt}}=R{\text{r}}_{{\text{N}}_{2}\text{O}}+T{\text{r}}_{{\text{N}}_{2}}$$ (25)

(molN h^-1^)

where $R{\text{r}}_{{\text{NO}}_{x}}$ (molN h^-1^) is the relevant $NO_{x}^{-}$/NO_x_ reduction rate, and $T{\text{r}}_{{\text{N}}_{\text{X}}}$ represents the gas transport rate between aqua and the headspace (Eq 29; N.B. $T{\text{r}}_{{\text{N}}_{\text{X}}}$< 0 for the net transport from aqua to the headspace).

The reduction of NO to N_2_O (Rr_NO_) and N_2_O to N_2_ ($R{\text{r}}_{{\text{N}}_{2}\text{O}}$) is modelled likewise as a function of the number of relevant cells and the velocity of e^-^-flow to NO and N_2_O (mol e^-^ cell^-1^ h^-1^), respectively:

$$R{\text{r}}_{\text{NO}}=\left({\text{Z}}^{\text{NaNi}}+{\text{Z}}^{\text{Ni}}\right)\times v_{\text{NO}}$$ (26)

$$R{\text{r}}_{{\text{N}}_{2}\text{O}}=\left(Z^{-}+{\text{Z}}^{\text{Na}}+{\text{Z}}^{\text{NaNi}}+{\text{Z}}^{\text{Ni}}\right)\times v_{{\text{N}}_{2}\text{O}}$$ (27)

(molN h^-1^)

where *v*_NO_ and $v_{{\text{N}}_{2}\text{O}}$ are obtained by the velocity of e^-^-flow to NO and N_2_O, respectively $\left(1\frac{\text{mol N}}{\text{mol}\;{\text{e}}^{-}}\right)$. $v{\text{e}}_{N_{2}O}^{-}$ is modelled as a Michaelis-Menten function of [N_2_O]_aq_, similarly as that of O_2_, ${\mathrm{NO}}_{3}^{-}$, and ${\mathrm{NO}}_{2}^{-}$ (Eqs 17 and 20), but $v{\text{e}}_{{}_{\text{NO}}}^{-}$ is modelled assuming a cooperative binding of two NO molecules with *c*Nor to form N_2_O [32]:

$$v{\text{e}}_{\text{NO}}^{-}=\frac{v{\text{e}}_{max\text{NO}}^{-}}{1+K_{2\mathrm{NO}}\left(\frac{1}{{\left[\text{NO}\right]}_{\mathrm{aq}}}+\frac{K_{1\mathrm{NO}}}{{\left[\text{NO}\right]}_{\mathrm{aq}}{}^{2}}\right)}$$ (28)

(mol cell^-1^ h^-1^)

where $v{\text{e}}_{max\text{NO}}^{-}$ (mol e^-^ cell^-1^ h^-1^) is the empirically determined maximum velocity of e^-^-flow to NO per cell, [NO]_aq_ (mol L^-1^) is the NO concentration in the liquid-phase, and K_1NO_ & K_2NO_ (mol L^-1^) are the equilibrium dissociation constants for the *c*Nor/NO- and *c*Nor/(NO)_2_ complex, respectively.

The transport of NO, N_2_O, and N_2_between the liquid and the headspace (Eqs 23–25) is modelled as:

$${\text{Tr}}_{\text{N}}={\text{k}}_{\text{t}}\times \left({\text{k}}_{\text{H}\left(\text{N}\right)}\times {\text{P}}_{\text{N}}-{\left[\text{N}\right]}_{\mathrm{aq}}\right)$$ (29)

(molN h^-1^)

where k_t_ is the empirically determined coefficient for the transport of each gas between the headspace and the liquid, k_H(N)_ (molN L^-1^ atm^-1^) is the solubility of NO, N_2_O, or N_2_ in water at 20°C, P_N_ (= [N]_g_×R×T, atm) is the partial pressure of each gas in the headspace, and [N]_aq_ (mol L^-1^) represents the concentration of each gas in the liquid-phase.

The amount of NO and N_2_O in the headspace (${\text{NO}}_{{\text{x}}_{\text{g}}}$, molN, Fig 2C) is a function of transport (Eq 29) and the disturbance by gas sampling. The latter is simulated as discrete events at time-points given as input to the model (equivalent to the sampling times in the experiment):

$$\Delta NO_{x(\text{S})}=\frac{{\text{NO}}_{{\text{x}}_{g}}\times \text{D}}{{\text{t}}_{\text{s}}}$$ (30)

(molN h^-1^)

where ΔNO_x(S)_ is the net change in the amount of ${\text{NO}}_{{\text{x}}_{g}}$ (molN), D (dilution) is the fraction of each gas removed and replaced by equal amount of He, and t_s_ (h) is the time taken to complete each sampling. For N_2_, the model ignores the sampling loss because the N_2_ production data to be compared with the model output are already corrected for the sampling disturbance [30]. Thus, the model estimates somewhat higher N_2_ concentrations than that experienced by the organisms, which is acceptable, since the concentration of N_2_ is unlikely to have consequences for the metabolism.

### Parameterisation

Most of the parameter values used in the model are well established in the literature (see Table 2); however, uncertain parameters include ${\text{K}}_{m{\text{O}}_{2}}$, ${\text{K}}_{m{\text{N}}_{2}\text{O}}$, $v{\text{e}}_{maxO_{2}}^{-}$, and $v{\text{e}}_{min}^{-}$.

**Table 2:** Model parameters.

		Description	Value	Units	Reference
**Butyrate treatments**					
$v{\text{e}}_{max\text{TCA}}^{-}$		Max. cell-specific rate of e^-^-delivery from the TCA cycle	1×10^14^	mol e^-^ cell^-1^ h^-1^	[18]
$v{\text{e}}_{maxO_{2}}^{-}$		The maximum cell-specific velocity of e^-^-flow to O_2_	4.22×10^−15^	mol e^-^ cell^-1^ h^-1^	Optimisation
$v{\text{e}}_{max{\text{NO}}_{3}^{-}}^{-}$		The maximum cell-specific velocity of e^-^-flow to ${\mathrm{NO}}_{3}^{-}$	1×10^−14^	mol e^-^ cell^-1^ h^-1^	[18]
$v{\text{e}}_{max{\text{NO}}_{2}^{-}}^{-}$		The maximum cell-specific velocity of e^-^-flow to ${\mathrm{NO}}_{2}^{-}$	2.65×10^−15^	mol e^-^ cell^-1^ h^-1^	[18]
$v{\text{e}}_{min}^{-}$		The min. velocity of e^-^-flow to O_2_/$NO_{x}^{-}$/NO_x_ required for protein synthesis (ATP)	1.87×10^−17^	mol e^-^ cell^-1^ h^-1^	Assumption
${\text{Ye}}_{{}_{{\text{O}}_{2}}}^{-}$		The growth yield per mole of electrons transferred to O_2_	2.74×10^13^	cells (mol e^-^)^-1^	[18]
${\text{Ye}}_{{}_{NO_{x}}}^{-}$		The growth yield per mole e^-^ to ${\mathrm{NO}}_{3}^{-}$, ${\mathrm{NO}}_{2}^{-}$, NO, or N_2_O	1.12×10^13^	cells (mol e^-^)^-1^	[18]
**Succinate treatments**					
$v{\text{e}}_{max\text{TCA}}^{-}$		Max. cell-specific rate of e^-^-delivery from the TCA cycle	9.34×10^−15^	mol e^-^ cell^-1^ h^-1^	[18]
$v{\text{e}}_{max{\text{O}}_{2}}^{-}$		The maximum cell-specific velocity of e^-^-flow to O_2_	4.42×10^−15^	mol e^-^ cell^-1^ h^-1^	[18]
$v{\text{e}}_{max{\text{NO}}_{3}^{-}}^{-}$		The maximum cell-specific velocity of e^-^-flow to ${\mathrm{NO}}_{3}^{-}$	9.34×10^−15^	mol e^-^ cell^-1^ h^-1^	[18]
$v{\text{e}}_{max{\text{NO}}_{2}^{-}}^{-}$		The maximum cell-specific velocity of e^-^-flow to ${\mathrm{NO}}_{2}^{-}$	2.01×10^−15^	mol e^-^ cell^-1^ h^-1^	[18]
$v{\text{e}}_{min}^{-}$		The minimum velocity of e^-^-flow to O_2_/$NO_{x}^{-}$/NO_x_ required for protein synthesis (ATP)	1.95×10^−17^	mol e^-^ cell^-1^ h^-1^	Assumption
${\text{Ye}}_{{}_{{\text{O}}_{2}}}^{-}$		The growth yield per mole of electrons transferred to O_2_	4.97×10^13^	cells (mol e^-^)^-1^	[18]
${\text{Ye}}_{{}_{NO_{x}}}^{-}$		The growth yield per mole e^-^ to ${\mathrm{NO}}_{3}^{-}$, ${\mathrm{NO}}_{2}^{-}$, NO, or N_2_O	1.52×10^13^	cells (mol e^-^)^-1^	[18]
**Parameters common to both succinate and butyrate treatments**					
[O_2_]_*na*_	The [O_2_] in aqua below which Nar production triggers		5.95×10^−5^	mol L^-1^	[18]
[O_2_]_*ni*_	The [O_2_] in aqua below which NirS production triggers		9.75×10^−6^	mol L^-1^	[18]
r_Na_	The specific-probability for Nar production		0.035	h^-1^	Optimisation
r_Ni_	The specific-probability for NirS production		0.004	h^-1^	Optimisation
$v{\text{e}}_{max\text{NO}}^{-}$	The maximum cell-specific velocity of e^-^-flow to NO		3.56×10^−15^	mol e^-^ cell^-1^ h^-1^	[33]
$v{\text{e}}_{max{\text{N}}_{2}\text{O}}^{-}$	The maximum cell-specific velocity of e^-^-flow to N_2_O		5.5×10^−15^	mol e^-^ cell^-1^ h^-1^	[24]
${\text{K}}_{m{\text{O}}_{2}}$	The half-saturation constant for O_2_ reduction		2.25×10^−7^	mol L^-1^	Optimisation
${\text{K}}_{m{\text{NO}}_{3}^{-}}$	The half-saturation constant for ${\mathrm{NO}}_{3}^{-}$ reduction		5×10^−6^	mol L^-1^	[34,35]
${\text{K}}_{m{\text{NO}}_{2}^{-}}$	The half-saturation constant for ${\mathrm{NO}}_{2}^{-}$ reduction		4.13×10^−6^	mol L^-1^	[36,37]
K_1NO_	The equilibrium dissociation constant for *c*Nor/NO complex		8×10^−14^	mol L^-1^	[33]
K_2NO_	The equilibrium dissociation constant for *c*Nor/(NO)_2_ complex		34×10^−9^	mol L^-1^	[33]
${\text{K}}_{m{\text{N}}_{2}\text{O}}$	The half-saturation constant for N_2_O reduction		5.93×10^−7^	mol N_2_O-N L^-1^	Optimisation
D	Dilution (due to sampling): fraction of gas replaced by He		0.013–0.016	–	[18]
${\text{k}}_{\text{H}\left({\text{O}}_{2}\right)}$	Solubility of O_2_ in water at 20°C		0.0014	mol L^-1^ atm^-1^	[38]
k_H(NO)_	Solubility of NO at 20°C		0.0021	mol L^-1^ atm^-1^	[30]
${\text{k}}_{\text{H}\left({\text{N}}_{2}\text{O}\right)}$	Solubility of N_2_O at 20°C		0.056	mol N_2_O-N L^-1^ atm^-1^	[38]
${\text{k}}_{\text{H}\left({\text{N}}_{2}\right)}$	Solubility of N_2_ at 20°C		0.00035	mol N_2_-N L^-1^ atm^-1^	[38]
k_t_	The coeff. for gas transport between headspace and liquid		3.6	L vial^-1^ h^-1^	Measured
O_2leak_	O_2_ leakage into the vial during each sampling		2.92×10^−9^	mol	Measured
R	Universal gas constant		0.083	L atm K^-1^ mol^-1^	–
T	Temperature		293.15	K	[18]
t_s_	The time taken to complete each sampling		0.017	h	[30]
Vol_g_	Headspace volume		0.07	L	[18]
Vol_aq_	Aqueous-phase volume		0.05	L	[18]

$K_{mO_{2}}$ (Eq 17). *Pa*. *denitrificans* has three haem-copper terminal oxidoreductases [39] with $K_{m{\text{O}}_{2}}$ ranging from nM to µM [40,41], so we decided to estimate the parameter value by optimising $K_{m{\text{O}}_{2}}$ for the low [O_2_] treatments data. Vensim was used for the optimisation, where $K_{m{\text{O}}_{2}}$ = 2.25×10^−7^ neatly simulated the O_2_ depletion for both the succinate- and butyrate-supplemented treatments.

**$K_{mN_{2}O}$.** *In vitro* studies of NosZ from *Pa*. *denitrificans* estimate the values for ${\text{K}}_{m{\text{N}}_{2}\text{O}}$ = 5 μM at 22°C and pH 7.1 [42] and 6.7 μM at 25°C and pH 7.1 [43]. When our model was simulated with ${\text{K}}_{m{\text{N}}_{2}\text{O}}$ in this range, given our empirically estimated $v{\text{e}}_{max{\text{N}}_{2}\text{O}}^{-}$ [24], the simulated N_2_O reached concentrations much higher than that measured (see Results/Discussion). A more adequate parameter value (= 0.6 μM) was found by optimising ${\text{K}}_{m{\text{N}}_{2}\text{O}}$ in Vensim. The value is within the range determined for soil bacterial communities [44].

$ve_{\max O_{2}}^{-}$ (Eq 17) could be estimated using the empirically determined cell yield per mole of electrons to O_2_ (${\text{Ye}}_{{}_{{\text{O}}_{2}}}^{-}$, cells per mol e^-^) and the maximum specific growth rate (μ, h^-1^): $v{\text{e}}_{max{\text{O}}_{2}}^{-}=\frac{\mu }{{\text{Ye}}_{{\text{O}}_{2}}^{-}}$. We are confident about the yields for the two C-substrates used, but the empirically determined μ for the butyrate treatments is suspiciously low (= 0.067 h^-1^), providing $v{\text{e}}_{max{\text{O}}_{2}}^{-}$ = 2.45×10^−15^ mol e^-^ cell^-1^ h^-1^. Simulations with this value grossly underestimated the rate of O_2_ depletion compared to measured, which forced us to estimate the parameter value by optimisation: $v{\text{e}}_{max{\text{O}}_{2}}^{-}$ = 4.42×10^−15^ and 4.22×10^−15^ mol e^-^ cell^-1^ h^-1^ for the succinate- and butyrate treatments, respectively. These values give μ = 0.22 and 0.12 h^-1^, respectively: for the succinate treatments, the value is very close to that empirically determined (= 0.2 h^-1^); for the butyrate treatments, the value seems more realistic than 0.067 h^-1^.

$ve_{\min}^{-}$ (Eqs 2, 5 and 7) is the per cell velocity of e^-^-flow to O_2_ ($v{\text{e}}_{{\text{O}}_{2}}^{-}$) assumed to generate minimum ATP required for synthesising the initial molecules of denitrification enzymes. Since we lack any empirical or other estimations for this parameter, it is arbitrarily assumed to be the $v{\text{e}}_{{\text{O}}_{2}}^{-}$ when [O_2_]_aq_ reaches 1 nM. At this concentration, $v{\text{e}}_{min}^{-}$ is determined by the Michaelis-Menten equation $\left(v{\text{e}}_{min}^{-}=\frac{v{\text{e}}_{max{\text{O}}_{2}}^{-}\times {{[\text{O}}_{2}]}_{\text{aq}}}{{(\text{K}}_{m{\text{O}}_{2}}+{{[\text{O}}_{2}]}_{\text{aq}})}\right)$, using $v{\text{e}}_{max{\text{O}}_{2}}^{-}$ and ${\text{K}}_{m{\text{O}}_{2}}$ given above. The values obtained for the succinate- and butyrate-supplemented treatments = 1.96×10^−17^ and 1.87×10^−17^ mol e^-^ cell^-1^ h^-1^, respectively, which for both the cases is 0.44% of $v{\text{e}}_{max{\text{O}}_{2}}^{-}$. To investigate the impact of $v{\text{e}}_{min}^{-}$ on the model behaviour (r_Na_ and r_Ni_, Eqs 1, 2, 4, 5, 6 and 7), sensitivity analyses were performed by simulating the model with $v{\text{e}}_{min}^{-}$ corresponding to [O_2_]_aq_ = 5×10^−9^, 5×10^−10^, and 0 mol L^- 1^ (see Results/Discussion).

## Results/Discussion

### Low probabilistic initiation of *nar* transcription, resulting in the fraction of the population with Nar < 100%

**Fig 3:**
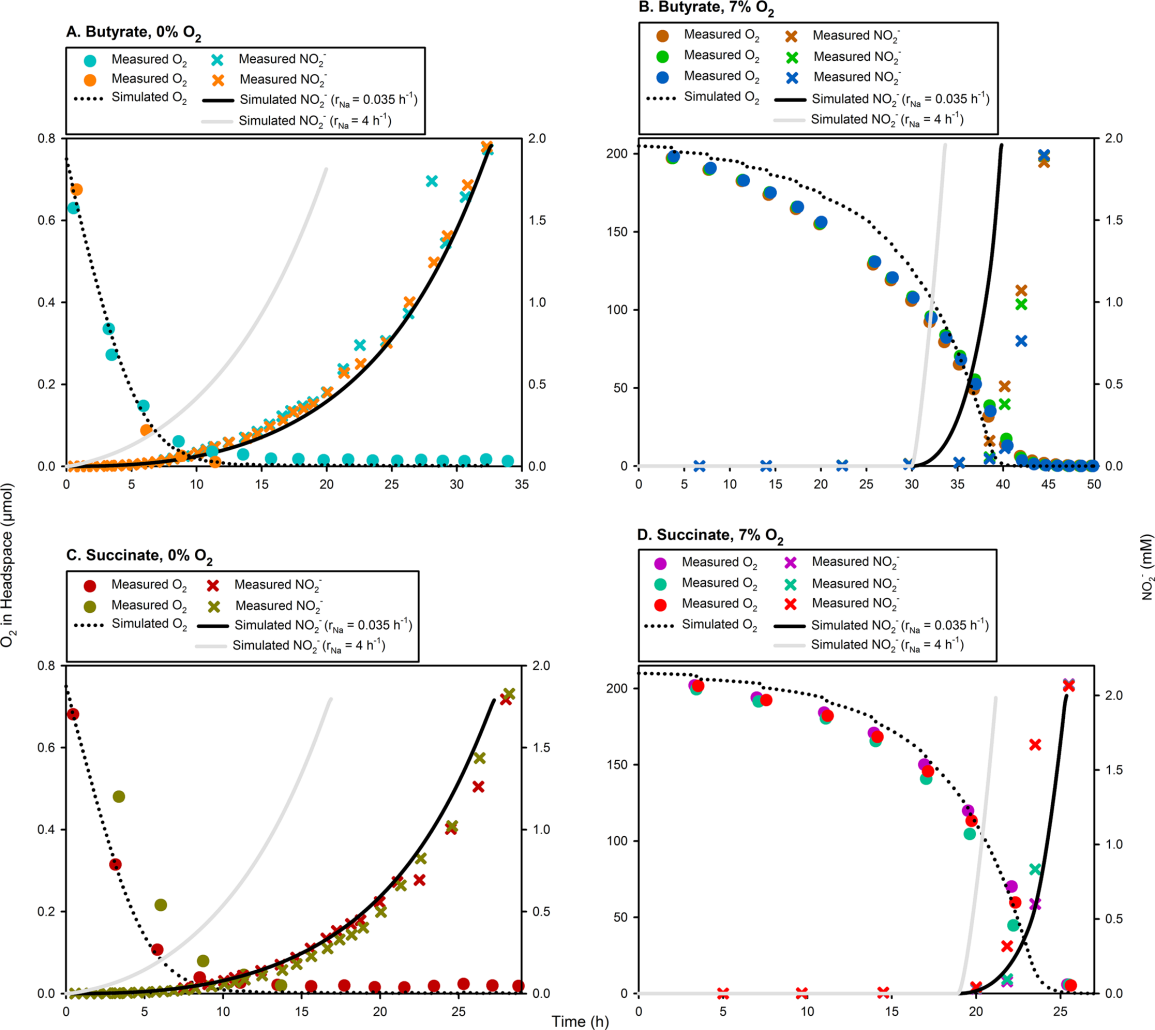
Comparison of measured and simulated ${\mathrm{NO}}_{2}^{-}$ accumulation assuming definitive versus stochastic initiation of *nar* transcription. To test the assumption of a single homogeneous population with almost all cells expressing *nar* in response to O_2_ depletion, we forced our model to achieve 98% Nar-positive cells (Z^Na^) within an hour by setting the specific-probability of initiating *nar* transcription (r_Na_) = 4 h^-1^. This resulted in grossly overestimated rates of ${\mathrm{NO}}_{2}^{-}$ accumulation for all treatments (grey curves). In contrast, we simulated the model with r_Na_ = 0.035 h^-1^ obtained through optimisation, resulting in a reasonable agreement with measurements for all treatments (except for an apparent time frameshift for the Butyrate, 7% O_2_ treatment).

To test the assumption of a single homogeneous population with all cells producing Nar in response to O_2_ depletion, we simulated the model with the specific probability for a Z^−^ cell to initiate *nar* transcription (r_Na_) = 4 h^-1^. This resulted in 98% of the cells possessing Nar within an hour (see Eqs 1–3). Evidence suggests that less than half an hour is required to synthesise denitrification enzymes [17,18], but an hour’s time is assumed here to allow margin for error. The results show that, for all the treatments, the simulated ${\mathrm{NO}}_{2}^{-}$ production (mol vial^-1^) grossly overestimates that measured (Fig 3).

To find a reasonable parameter value, we optimised r_Na_ for the 0% O_2_ treatments, so that the simulated ${\mathrm{NO}}_{2}^{-}$ production matches that measured. The results (Table 3) suggest that a low probabilistic initiation of *nar* transcription (average r_Na_ = 0.035 h^-1^) is adequate to simulate the measured ${\mathrm{NO}}_{2}^{-}$ kinetics (Fig 3). In the Butyrate, 7% O_2_ treatment (Fig 3B), the simulated ${\mathrm{NO}}_{2}^{-}$ starts earlier, but the rate of accumulation is similar to that measured.

**Table 3:** Specific-probability of *nar* and *nirS* transcriptional initiation (r_Na_ and r_Ni_, respectively) estimated for each treatment by optimisation (best match between the simulated and measured data).

Batch	C-source	Treatment*: O_2_ (vol.%), ${\mathrm{NO}}_{3}^{-}$ (mM)	Optimal r_Na_ (h^-1^)	Optimal r_Ni_ (h^-1^)
**1**	Butyrate	~0, 2	0.041	0.005
**2**	Butyrate	7, 2	–	0.004
**3**	Succinate	~0, 2	0.030	0.005
**4**	Succinate	7, 2	–	0.003
			**Avg. = 0.035**	**Avg. = 0.004**

*Treatment refers to the C-source, initial oxygen concentration in the headspace (measured as headspace-vol.%), and initial ${\text{NO}}_{3}^{-}$ concentration in the medium (mM).

Once O_2_ falls below a certain threshold, the production of Nar is assumed to trigger with r_Na_ = 0.035 h^-1^ and last until a minimum of respiration is sustained by the e^-^-flow to O_2_ and N_2_O ($v{\text{e}}_{{}_{{\text{O}}_{2}}}^{-}$ and $v{\text{e}}_{{}_{{\text{N}}_{2}\text{O}}}^{-}$), assumed to fulfil the ATP needs for Nar production (Eqs 1 and 2). But the production of Nar sustained by $v{\text{e}}_{{}_{{\text{N}}_{2}\text{O}}}^{-}$ was inconsequential for simulating the measured ${\mathrm{NO}}_{2}^{-}$ production, since ${\mathrm{NO}}_{3}^{-}$ was already exhausted when N_2_O started accumulating (i.e., when $v{\text{e}}_{{}_{{\text{N}}_{2}\text{O}}}^{-}$ > 0). For this reason, the fraction that produced Nar (F_Na_, Eq 3 and Table 4) is calculated as functional (= 0.23–0.43) and theoretical (= 0.56–0.81), where the first is the fraction actually responsible for ${\mathrm{NO}}_{2}^{-}$ production (sustained by $v{\text{e}}_{{}_{{\text{O}}_{2}}}^{-}$), but the latter also incorporates the fraction that produced Nar after the exhaustion of ${\mathrm{NO}}_{3}^{-}$ (sustained by $v{\text{e}}_{{}_{{\text{O}}_{2}}}^{-}$as well as $v{\text{e}}_{{}_{{\text{N}}_{2}\text{O}}}^{-}$). The rationale behind calculating the theoretical F_Na_ is the empirical data indicating that Nar transcription is not turned off in response to ${\mathrm{NO}}_{3}^{-}$ depletion [18]. Although our model cannot test the theoretical F_Na_, but the functional F_Na_ suggests that, contrary to the common assumption, the measured ${\mathrm{NO}}_{2}^{-}$ kinetics can be neatly explained by only 23–43.3% of the population producing Nar in response to O_2_ depletion.

**Table 4:** The fraction of the population with Nar (F_Na_) and NirS (F_Ni_) estimated based on the optimal specific-probability of *nar* and *nirS* transcriptional initiation (r_Na_ and r_Ni_), respectively.

Batch	C-source	O_2_ (vol.%), ${\mathrm{NO}}_{3}^{-}$ (mM)	Functional F_Na_ * (unitless)	Theoretical F_Na_ * (unitless)	F_Ni_ (unitless)
**1**	Butyrate	~0, 2	0.433	0.813	0.221
**2**	Butyrate	7, 2	0.343	0.656	0.088
**3**	Succinate	~0, 2	0.357	0.803	0.206
**4**	Succinate	7, 2	0.230	0.564	0.077

*Functional F_Na_ is the fraction of cells expressing Nar while ${\text{NO}}_{3}^{-}$ is still present, while Theoretical F_Na_ is the fraction expressing Nar when including the theoretical recruitment after ${\text{NO}}_{3}^{-}$ depletion (supported by energy from N_2_O reduction).

### Very low probabilistic initiation of *nirS* transcription

**Fig 4:**
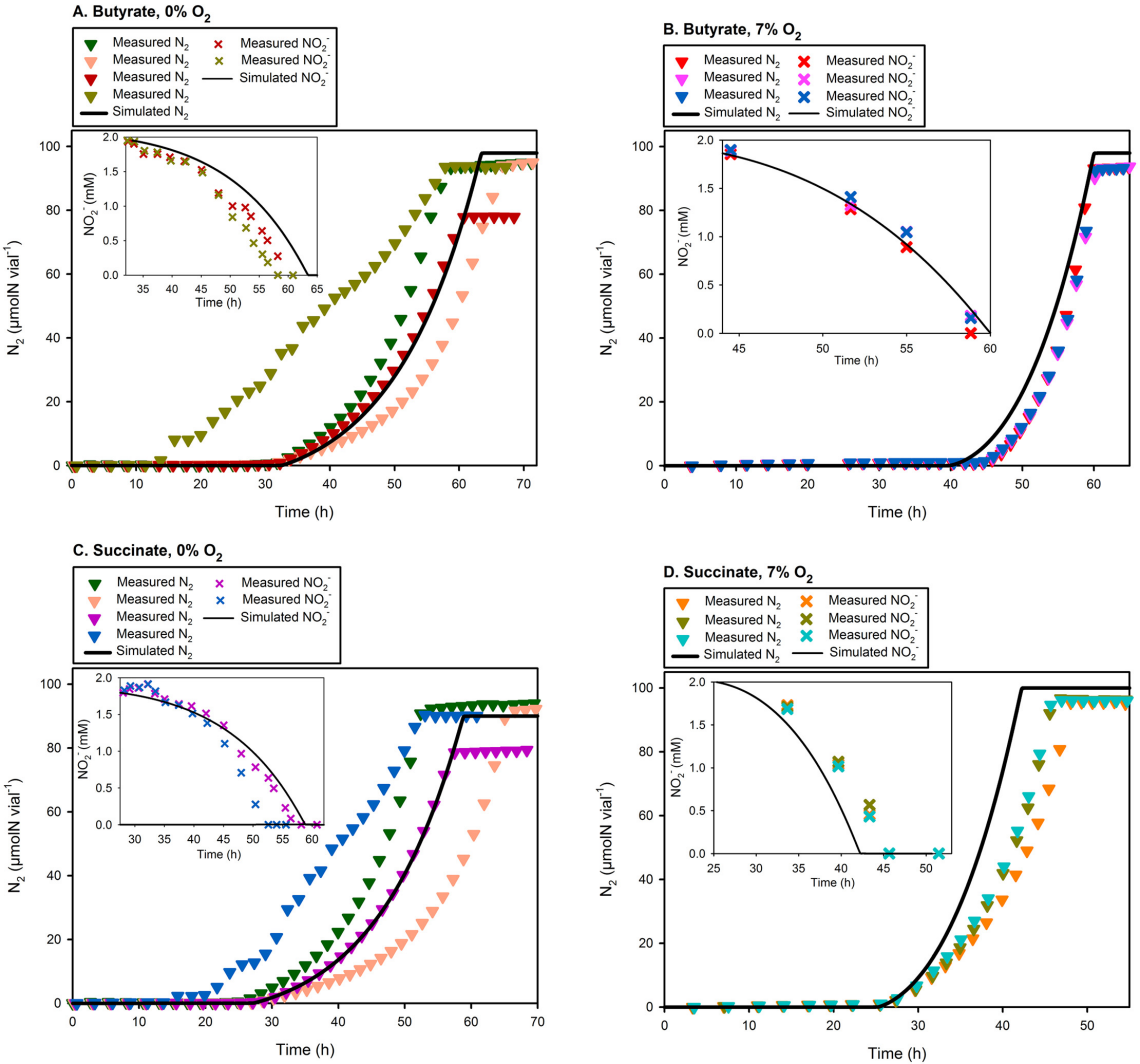
Comparison of measured and simulated data assuming stochastic initiation of *nirS* transcription. Each panel compares the measured ${\mathrm{NO}}_{2}^{-}$ depletion (sub-panel) and N_2_ accumulation (main panel; n = 3–4) with simulations. The simulations are carried out with an optimised specific-probability of *nirS* transcriptional initiation (average r_Ni_ = 0.004 h^-1^, Eqs 4, 5, 6 and 7), allowing 7.7–22.1% of the population to produce NirS + *c*Nor (Eq 8) during the available time-window (= 19.5–47.3 h).

When we optimised the specific probability of *nirS* transcriptional activation (r_Ni_, see Eqs 4, 5, 6 and 7) to fit the measured data, the average r_Ni_ = 0.004 h^-1^ (Table 3) adequately simulated the measured ${\mathrm{NO}}_{2}^{-}$ depletion and N_2_ accumulation (Fig 4). The recruitment to denitrification lasted for 19.5–47.3 h, i.e., the time when [O_2_] was below a critical concentration and the velocity of e^-^-flow to O_2_ and the relevant $NO_{x}^{-}$/NO_x_ remained above a critical minimum (Eqs 4, 5, 6 and 7). The resulting fraction recruited to denitrification (F_Ni_, see Eq 8 and Table 4) was 0.08–0.18, the bulk of which depended on the e^-^-flow to ${\mathrm{NO}}_{3}^{-}$ and N_2_O (instead of aerobic respiration).

To test whether the measured data could be explained without the recruitment sustained by ${\mathrm{NO}}_{3}^{-}$ and N_2_O respiration, we also simulated the model with the recruitment as a function of O_2_ alone and re-optimised r_Ni_, which on average increased to 0.012 h^-1^ (providing F_Ni_ = 0.083–0.35). This was expected since O_2_ is exhausted rather quickly, shrinking the time-window available for the recruitment. Comparatively, these simulations were less satisfactory: using the average r_Ni_ = 0.012 h^-1^ generally resulted in larger deviations than for the default simulations (S2 Fig), and the optimal r_Ni_ for individual treatments varied grossly (50% higher values for the ~0% O_2_ treatments than for the 7% O_2_ treatments). This contrasts the default simulations, where the optimal r_Ni_ values for individual treatments were quite similar.

### Sensitivity of r_Na_ and r_Ni_ to $v{\text{e}}_{min}^{-}$

Recruitment to denitrification (both *nar* and *nirS* transcription) is assumed to continue only as long as the combined e^-^-flow to O_2,_ ${\mathrm{NO}}_{3}^{-}$ and N_2_O is greater than $v{\text{e}}_{min}^{-}$ (Eqs 1, 2, 4, 5, 6 and 7). To test the model’s sensitivity to this parameter, we estimated r_Na_ and r_Ni_ by optimisation for different values of $v{\text{e}}_{min}^{-}$, relative to the default value = 1.95×10^−17^ mol e^-^ cell^-1^ h^-1^. For all cases, the model was able to adequately simulate the measured N_2_ kinetics by moderate adjustments of r_Na_ and r_Ni_. Table 5 shows the average optimal values of r_Na_ and r_Ni_, obtained by fitting the simulated N_2_ kinetics to the data, for different values of $v{\text{e}}_{min}^{-}$. S3 Fig shows adequate simulations of the measured N_2_ kinetics assuming $v{\text{e}}_{min}^{-}$ = 0, with optimised r_Na_ = 0.033 h^-1^ and r_Ni_ = 0.0033 h^-1^. Thus, although assuming $v{\text{e}}_{min}^{-}$ > 0 appears logical, it is not necessary to explain the measured data.

**Table 5:** Estimated r_Na_ and r_Ni_, depending on $v{\text{e}}_{min}^{-}$ a.

$v{\text{e}}_{min}^{-}$ (mol e^-^ cell h^-1^)	Optimal r_Na_ (h^-1^)	Optimal r_Ni_ (h^-1^)
**5 *×* Default** *	0.041	0.0062
**Default**	0.035	0.0041
**0.5 *×* Default**	0.034	0.0035
**0**	0.033	0.0033

*Refers to the default value = 1.95×10^−17^ mol e^-^ cell^-1^ h^-1^.

### N_2_O kinetics

**Fig 5:**
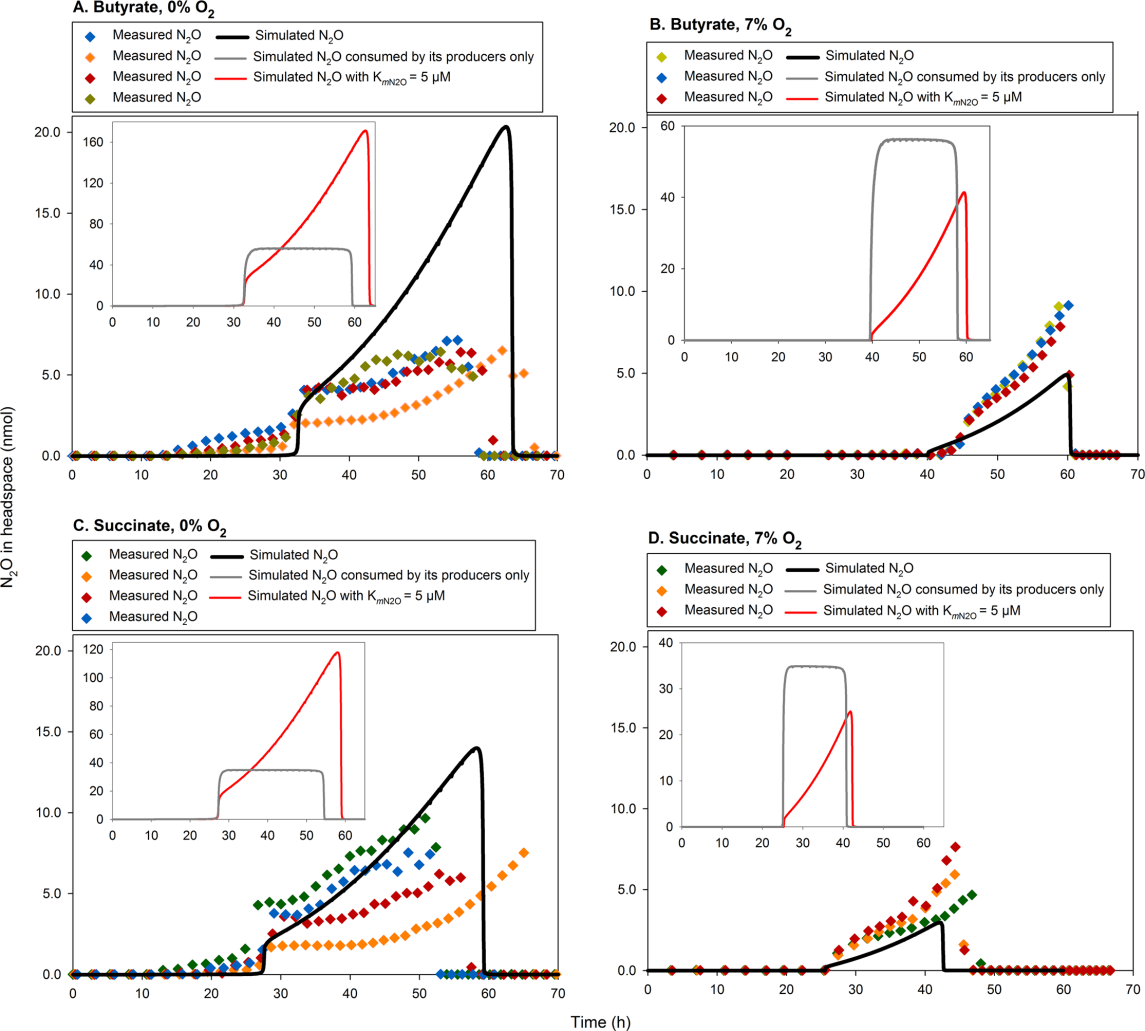
Comparison of the measured N_2_O with that simulated. Each main panel (A–D) compares the measured N_2_O (single vial results) with the default simulation using the parameter values given in Table 2, i.e., ${\text{K}}_{m{\text{N}}_{2}\text{O}}$ = 0.6 μM (estimated through optimisation) and $v{\text{e}}_{max{\text{N}}_{2}\text{O}}^{-}$ = 5.5×10^−15^ mol e^-^ cell^-1^ h^-1^ [24]. In contrast, each inserted panel shows the simulated N_2_O assuming 1) N_2_O consumption only by the cells producing N_2_O (Z^NaNi^ + Z^Ni^), and 2) the literature value for ${\text{K}}_{m{\text{N}}_{2}\text{O}}$ = 5 μM [42]. The results show that the default simulation best explains the measured N_2_O kinetics, assuming its production by a small fraction (Z^NaNi^ + Z^Ni^) and consumption by the entire population (Z^−^ + Z^Na^+ Z^NaNi^ + Z^Ni^).

To simulate the N_2_O kinetics, we used $v{\text{e}}_{max{\text{N}}_{2}\text{O}}^{-}$ = 5.5×10^−15^ mol e^-^ cell^-1^ h^-1^, empirically determined under similar experimental conditions as simulated here [24], and adopted the literature values for ${\text{K}}_{m{\text{N}}_{2}\text{O}}$ [= 5 and 7 μM 42,43, respectively]. But with ${\text{K}}_{m{\text{N}}_{2}\text{O}}$ = 5 μM, the model predicted N_2_O accumulation ~10–20 times higher than measured for the ~0% and ~2–3 times higher for the 7% O_2_ treatments (Fig 5). This forced us to simulate the model with the parameter value estimated by optimisation, providing the average ${\text{K}}_{m{\text{N}}_{2}\text{O}}$ = 0.6 μM.

The measured N_2_O shows a conspicuous increase throughout the entire active denitrification period, and this phenomenon is neatly captured by the model. The reason for this model prediction is that the number of N_2_O producing cells (Z^NaNi^ + Z^Ni^, Fig 2A) is low to begin with compared to the number of N_2_O consuming cells (Z^−^ + Z^Na^+ Z^NaNi^ + Z^Ni^), but the fraction of N_2_O producers will increase during the anoxic phase for two reasons: one is the recruitment to Z^NaNi^ & Z^Ni^, another is the fact that the model predicts approximately three times faster cell-specific growth rate for Z^NaNi^ & Z^Ni^ than for Z^−^ & Z^Na^ ($v{\text{e}}_{{\text{N}}_{2}\text{O}}^{-}$ is identical for all groups, while $v{\text{e}}_{{\text{NO}}_{2}^{-}}^{-}$ and $v{\text{e}}_{\text{NO}}^{-}$ are both zero for Z^−^ & Z^Na^ but for Z^NaNi^ & Z^Ni^, it holds that $v{\text{e}}_{{\text{NO}}_{2}^{-}}^{-}$ ≈ $v{\text{e}}_{\text{NO}}^{-}$ > $v{\text{e}}_{{\text{N}}_{2}\text{O}}^{-}$_._ To illustrate this phenomenon, we ran the model, assuming that the Z^−^ & Z^Na^ cells had no N_2_O reductase, resulting in *a)* constant N_2_O concentration throughout the entire anoxic phase and *b)* much higher N_2_O concentrations than measured (Fig 5). The overestimation is a trivial result, easily avoidable by increasing $v{\text{e}}_{max{\text{N}}_{2}\text{O}}^{-}$ or decreasing ${\text{K}}_{m{\text{N}}_{2}\text{O}}$ moderately. However, the prediction of a constant N_2_O concentration is clearly in conflict with the experimental data, and no parameterisation could force the model to reproduce this phenomenon other than the differential expression of denitrification genes.

Hence, although there is room for further refinements, our default assumption regarding differential expression of NirS and NosZ explains the observed N_2_O kinetics: 1) abrupt initial accumulation to very low levels due to recruitment of relatively small numbers to the N_2_O producing pools (Z^NaNi^ & Z^Ni^), and 2) increasing N_2_O concentration due to recruitment and faster cell-specific growth of Z^NaNi^ & Z^Ni^ than that of the cells only consuming N_2_O (Z^−^ + Z^Na^).

This modelling exercise sheds some light on the possible role of regulatory biology of denitrification in controlling N_2_O emissions from soils. If all cells in soils had the same regulatory phenotype as *Pa*. *denitrificans*, their emission of N_2_O would probably be miniscule, and soils could easily become strong net sinks for N_2_O because the majority of cells would be ‘truncated denitrifiers’ with only N_2_O reductase expressed. It remains to be tested, however, if the regulatory phenotype of *Pa*. *denitrificans* is a rare or a common phenomenon among full-fledged denitrifiers. We foresee that further exploration of denitrification phenotypes will unravel a plethora of response patterns.

### Conclusion

Using dynamic modelling, we have demonstrated that the denitrification kinetics in *Pa*. *denitrificans* can be adequately explained by assuming low probabilistic transcriptional activation of the *nar* and *nirS* genes and a subsequent autocatalytic expression of the enzymes. Such autocatalytic gene expressions are common in prokaryotes, rendering a population heterogeneous because of the stochastic initiation of gene transcription, with a low probability [45]. For N_2_O kinetics, our hypothesis was that *a)* the gas is produced by a fraction of the incubated population that is able to initiate *nirS* transcription with a certain probability, leading to a coordinated expression of *nirS* + *nor* via NO [16], and *b)* N_2_O is consumed by the entire population because, in response to anoxia, *nosZ* is readily induced by FnrP [24]. Our model corroborated this hypothesis by reasonably simulating the N_2_O kinetics with the specific-probability of *nirS* transcriptional activation = 0.004 h^-1^, resulting in 7.7–22.1% of the population producing NirS + *c*Nor (hence N_2_O), but all cells producing NosZ (hence equally consuming N_2_O).

## Supporting Information

S1 Dynamic Model The folder contains the dynamic model used in this study ‘Hassan_et_al_2015_Pa._denitrificans.mdl’. The model requires Vensim (Double Precision), which is available at http://vensim.com/free-download/. The zip folder also contains files with the empirical data; these files are automatically loaded into the model when it is run.
(ZIP)

S1 Fig
*Pa*. *denitrificans* gas and ${\mathrm{NO}}_{2}^{-}$ kinetics.Typical gas kinetics (O_2_, NO, N_2_O, N_2_) and ${\mathrm{NO}}_{2}^{-}$ accumulation in *Pa*. *denitrificans* during the transition from aerobic respiration to denitrification; batch cultures, n = 3; 20°C; Sistrom’s medium; 2 mM KNO_3_ and 7 vol% initial O_2_ in the headspace. All the available ${\mathrm{NO}}_{3}^{-}$ (100 μmol vial^-1^) was recovered as ${\mathrm{NO}}_{2}^{-}$ before the onset of N-gas production. In previous experiments [17], N_2_O concentrations were below the detection limit of the system, but thanks to a new system with electron capture detector, the N_2_O kinetics were monitored with reasonable precision. Adapted from [18].(TIF)

S2 FigComparison of measured and simulated data assuming stochastic initiation of *nirS* transcription with aerobic respiration being the only energy source for producing NirS + *c*Nor.In each panel, the measured ${\mathrm{NO}}_{2}^{-}$ depletion (sub-panel) and N_2_ accumulation (main panel; n = 3–4) are compared with simulations. The simulations here are to be compared with the default simulations (Fig 4), which were run assuming that the coordinated NirS + *c*Nor production (via *nirS* transcriptional activation) is sustained by the energy generated by O_2_ as well as ${\text{NO}}_{3}^{-}$ and/or N_2_O reduction. The default simulations provided an average specific-probability of *nirS* transcriptional activation (r_Ni_) = 0.004 h^-1^ (Eqs 4, 5, 6 and 7) by optimisation, allowing 7.7–22.1% of the population to produce NirS + *c*Nor (Eq 8) in 19.5–47.3 h. To match the measured data here, the average r_Ni_ had to be raised to 0.012 h^-1^, since the time available for the enzyme synthesis shrank (= 3.5–16 h) due to a rapid exhaustion of O_2_. Comparatively, the assumption that the ATP from ${\text{NO}}_{3}^{-}$ and/or N_2_O reduction should help cells produce denitrification enzymes seems more plausible and provide better agreement with the measured data.(TIF)

S3 FigMeasured vs. simulated N_2_ kinetics assuming $v{\text{e}}_{min}^{-}$ = 0.The default simulations are carried out assuming that for a cell to produce first molecules of Nar and NirS, a minimum of e^-^-flow to an available e^-^-acceptor ($v{\text{e}}_{min}^{-}$, mol e^-^ cell^-1^ h^-1^) is necessary to generate a minimum of ATP required for protein synthesis (Eqs 1, 2, 4, 5, 6 and 7). Although assuming $v{\text{e}}_{min}^{-}$ > 0 seems logical, the measured N_2_ kinetics are adequately simulated here with $v{\text{e}}_{min}^{-}$ = 0. This shows that the assumption is not necessary to explain the measured data.(TIF)
