# Supplementary figures and images for: Transient Accumulation of NO2 - and N2O during Denitrification Explained by Assuming Cell Diversification by Stochastic Transcription of Denitrification Genes
Source: PLoS Comput Biol. 2016 Jan 5;12(1):e1004621. doi: 10.1371/journal.pcbi.1004621 (PMC4701171; doi:10.1371/journal.pcbi.1004621)

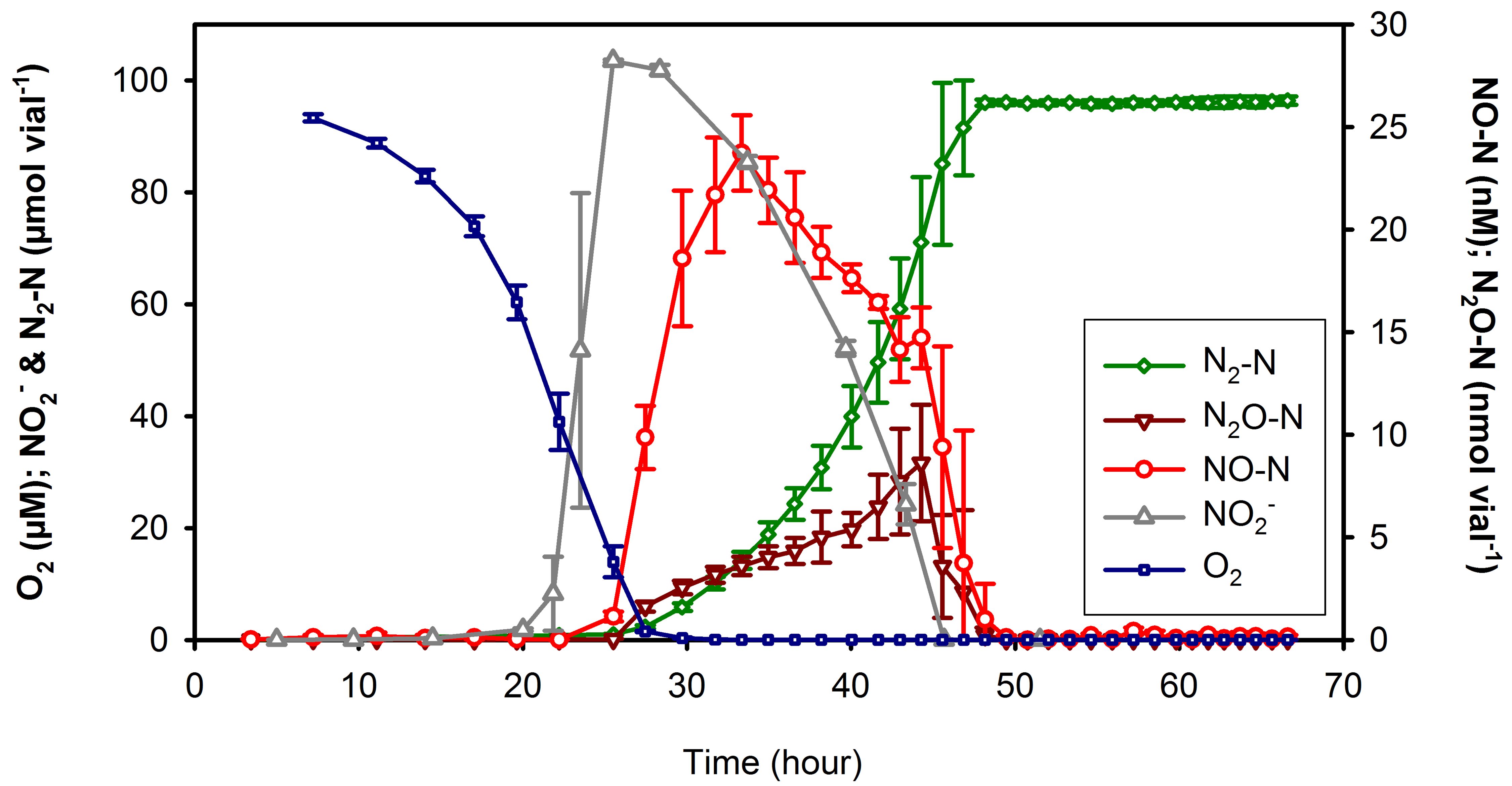

Supplement: S1 Fig — Typical gas kinetics (O2, NO, N2O, N2) and NO2− accumulation in Pa. denitrificans during the transition from aerobic respiration to denitrification; batch cultures, n = 3; 20°C; Sistrom’s medium; 2 mM KNO3 and 7 vol% initial O2 in the headspace. All the available NO3− (100 μmol vial-1) was recovered as NO2− before the onset of N-gas production. In previous experiments [17], N2O concentrations were below the detection limit of the system, but thanks to a new system with electron capture detector, the N2O kinetics were monitored with reasonable precision. Adapted from [18]. (TIF) [file pcbi.1004621.s002.tif]

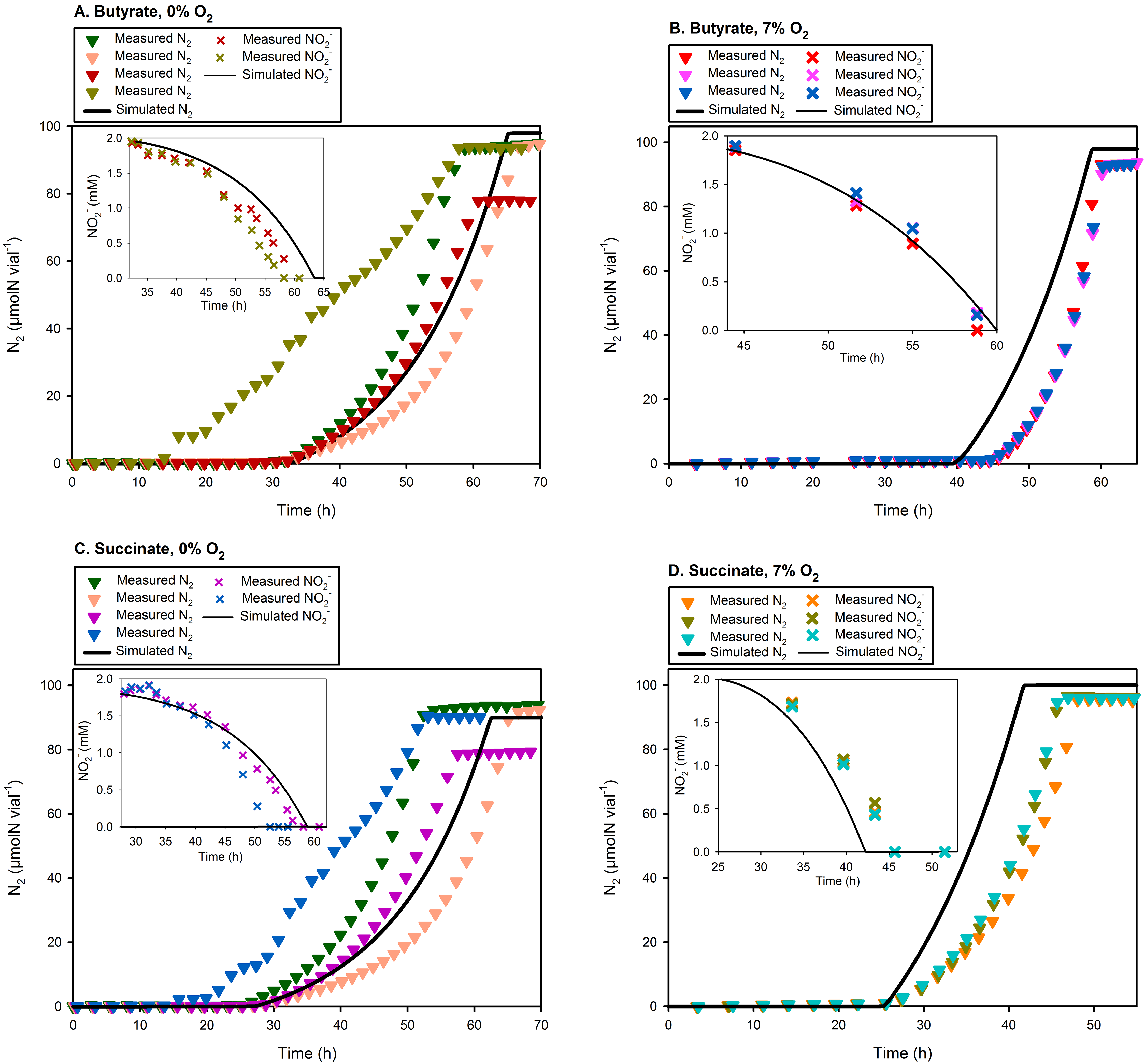

Supplement: S2 Fig — In each panel, the measured NO2− depletion (sub-panel) and N2 accumulation (main panel; n = 3–4) are compared with simulations. The simulations here are to be compared with the default simulations (Fig 4), which were run assuming that the coordinated NirS + cNor production (via nirS transcriptional activation) is sustained by the energy generated by O2 as well as NO3− and/or N2O reduction. The default simulations provided an average specific-probability of nirS transcriptional activation (rNi) = 0.004 h-1 (Eqs 4, 5, 6 and 7) by optimisation, allowing 7.7–22.1% of the population to produce NirS + cNor (Eq 8) in 19.5–47.3 h. To match the measured data here, the average rNi had to be raised to 0.012 h-1, since the time available for the enzyme synthesis shrank (= 3.5–16 h) due to a rapid exhaustion of O2. Comparatively, the assumption that the ATP from NO3− and/or N2O reduction should help cells produce denitrification enzymes seems more plausible and provide better agreement with the measured data. (TIF) [file pcbi.1004621.s003.tif]

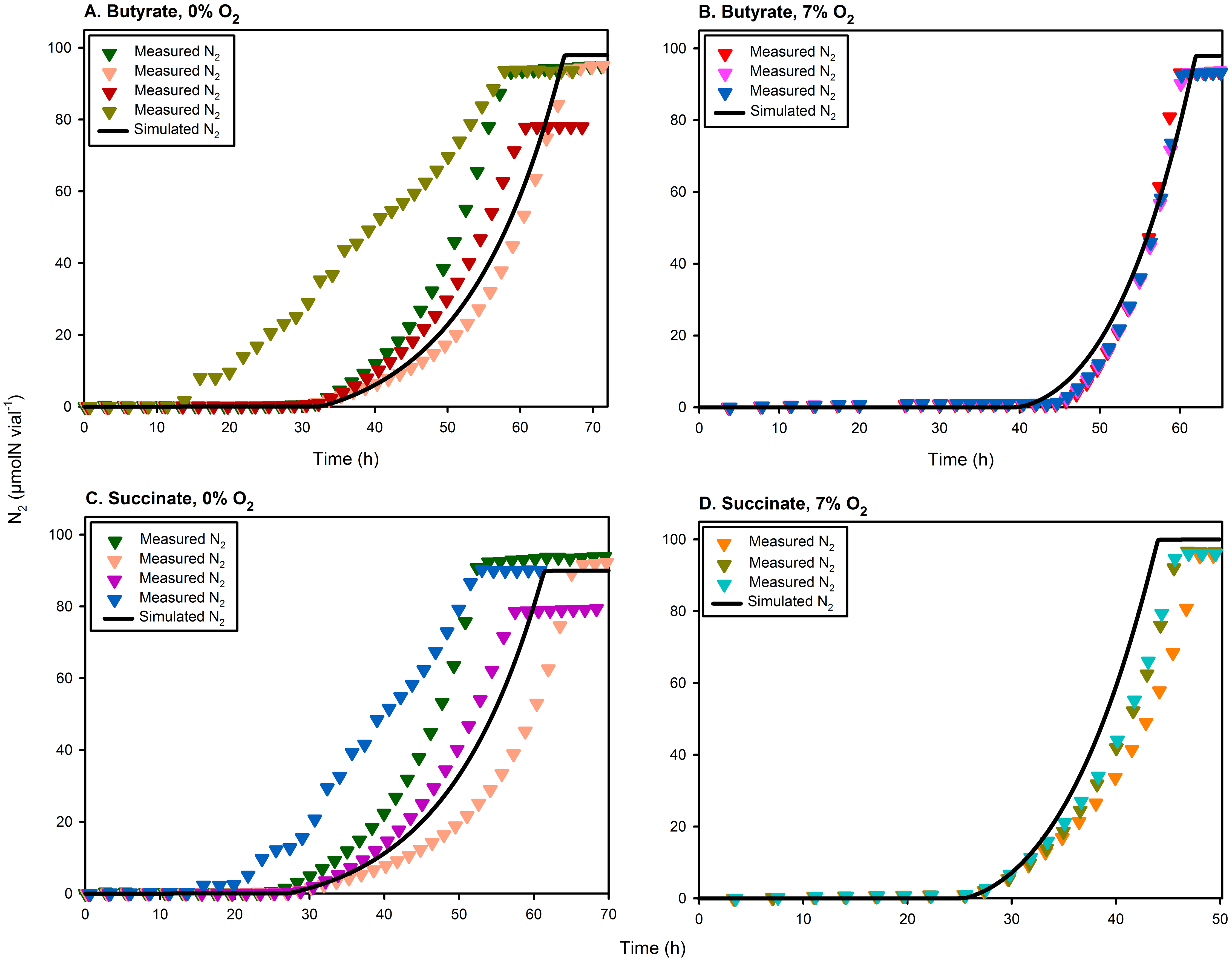

Supplement: S3 Fig — The default simulations are carried out assuming that for a cell to produce first molecules of Nar and NirS, a minimum of e--flow to an available e--acceptor (vemin−, mol e- cell-1 h-1) is necessary to generate a minimum of ATP required for protein synthesis (Eqs 1, 2, 4, 5, 6 and 7). Although assuming vemin− > 0 seems logical, the measured N2 kinetics are adequately simulated here with vemin− = 0. This shows that the assumption is not necessary to explain the measured data. (TIF) [file pcbi.1004621.s004.TIF]
